# A Tradeoff Between the Escape from *N*′-Mediated Resistance and Virulence in Pepper Mild Mottle Virus Through Reduced Virus Accumulation

**DOI:** 10.3390/plants14162471

**Published:** 2025-08-09

**Authors:** Hamdayanty, Kengo Idehara, Ken-Taro Sekine, Go Atsumi, Reiko Sekine, Chika Tateda, Takashi Yaeno, Hidetaka Kaya, Kappei Kobayashi

**Affiliations:** 1 The United Graduate School of Agricultural Sciences, Ehime University, Matsuyama 790-8566, Ehime, Japan; hamdaptn@gmail.com (H.); yaeno.takashi.rv@ehime-u.ac.jp (T.Y.); kaya.hidetaka.hu@ehime-u.ac.jp (H.K.); 2 Department of Plant Pest and Disease, Faculty of Agriculture, Hasanuddin University, Makassar 90245, South Sulawesi, Indonesia; 3 Graduate School of Agriculture, Ehime University, Matsuyama 790-8566, Ehime, Japan; 4 Iwate Biotechnology Research Center, Kitakami 024-0003, Iwate, Japan; k-sekine@cs.u-ryukyu.ac.jp (K.-T.S.); go-atsumi@aist.go.jp (G.A.); rsekine516@gmail.com (R.S.); ctateda@iwate-u.ac.jp (C.T.)

**Keywords:** coat protein, *N*′, mutagenesis, pepper mild mottle virus, resistance, tradeoff, virulence

## Abstract

*N*′ resistance is intrinsically broken by tobacco mosaic virus but is still effective against pepper mild mottle virus (PMMoV), including those breaking *L* resistance in peppers. To evaluate the durability of *N*′ resistance to PMMoV, we performed random mutagenesis of the coat protein (CP) gene of PMMoV. We isolated 11 CP mutants with two to six amino acid changes that escaped the *N*′-mediated resistance response in *Nicotiana sylvestris*. Some mutants and their derivatives, which had minimal mutations to escape *N*′-mediated resistance, exhibited reduced accumulation in inoculated leaves and loss of systemic infectivity in a susceptible pepper (*Capsicum annuum*) cultivar, as determined by RT-PCR analysis. Although the mutant CPs also escaped recognition by *L*^3^ and *L*^4^ resistance proteins from pepper in transient expression assays, the loss of systemic infectivity suggests that the mutants are unlikely to overcome *L*-mediated resistance. In *Nicotiana benthamiana*, a highly susceptible systemic host of PMMoV, ELISA and RT-qPCR indicated that the mutants consistently infected the host systemically, albeit with attenuated virulence and reduced virus accumulation, especially in younger leaves. The results collectively suggest that the reduced virus accumulation enabled the mutant PMMoV to escape *N*′-mediated resistance, and as a trade-off, compromised its virulence. The results also suggest that PMMoV CP modulates the systemic symptoms.

## 1. Introduction

The use of resistant cultivars for plant disease control is one of the ideal strategies because it is economical; labor-saving; and, more importantly, effective, unless resistance-breaking pathogens emerge (for a review, see [1]). Unfortunately, such pathogen strains often emerge, which may cause severe losses in the field. Resistance-breaking pathogens continuously threaten crop production until a new effective resistance gene (*R*-gene) is introduced into the crop cultivars. However, the development of new *R*-genes is difficult, and only one study has artificially generated an *R*-gene with a broader spectrum [2]. A possible solution would be to use an R-gene from different crop species.

Tobamovirus species are among the most important pathogens affecting crops, including pepper. In peppers, *L* gene alleles provide resistance to tobamovirus species [3,4,5]. We previously cloned them and revealed that they encode a typical resistance protein (R-protein) with coiled-coil (CC), nucleotide-binding (NB), and leucine-rich repeat (LRR) domains [6]. The seven alleles of a single copy *L* gene, namely *L*^1^, *L*^1*a*^, *L*^1*c*^, *L*^2^, *L*^2*b*^, *L*^3^, and *L*^4^, encode protein products that recognize tobamovirus coat protein (CP) [6,7,8,9,10] to trigger resistance response accompanying hypersensitive reaction (HR), a rapid cell death at the infection site. These alleles have been functionally categorized into either of the classic nomenclatures, *L*^1^, *L*^2^, *L*^3^*,* and *L*^4^, of which the latter two confer resistance to pepper mild mottle virus (PMMoV). PMMoV strains that break the *L*^3^ resistance have one or two amino acid substitutions in CP, which can still elicit *L*^4^ resistance [7,11,12,13,14]. However, other PMMoV strains have broken *L*^4^ resistance [15,16]. Thus, there is no effective *R*-gene for all pepper-infecting tobamovirus species available in pepper crops.

As an alternative to the broken *L* gene, *R*-genes from other crops can be used to protect pepper crops from tobamovirus infection. A possible candidate is the *N*′ gene, which shares the viral avirulence (Avr) factor, tobamovirus CPs, with the *L* gene [17,18] and encodes a CC-NB-LRR protein highly homologous to the L protein [19]. Any L gene-breaking PMMoV strains have not broken the *N*′ gene, although it does not confer resistance to tobacco mosaic virus (TMV). In contrast, although several PMMoV strains have easily broken the L gene, it has been durable against TMV [20]. Therefore, combining the *N*′ and *L* genes is expected to provide robust resistance to any tobamovirus species. However, a possible pitfall of this strategy is that a “super strain” (an analogy to “super race” often mentioned for microbial pathogens [21]) of PMMoV, which simultaneously breaks both *N*′ and *L* genes, could emerge.

To assess the risk of the emergence of the “super strain”, we established an experimental system to evaluate the durability of the *N*′ gene to PMMoV, in which random mutations were introduced into the CP genes of a PMMoV clone for agroinfection [22]. The resulting mutants were tested for their ability to escape *N*′-mediated resistance [23]. We identified 11 mutants out of approximately 400 clones tested that escaped *N*′-mediated resistance. The mutants were further characterized in susceptible host plants. Here, we demonstrate that the mutations required for escaping *N*′-mediated resistance severely affect virus accumulation in both inoculated and uninoculated upper leaves, and thereby, the virulence of the mutant viruses.

## 2. Results

### 2.1. Isolation of PMMoV Mutants Escaping N′-Mediated Resistance

**Figure 1:**
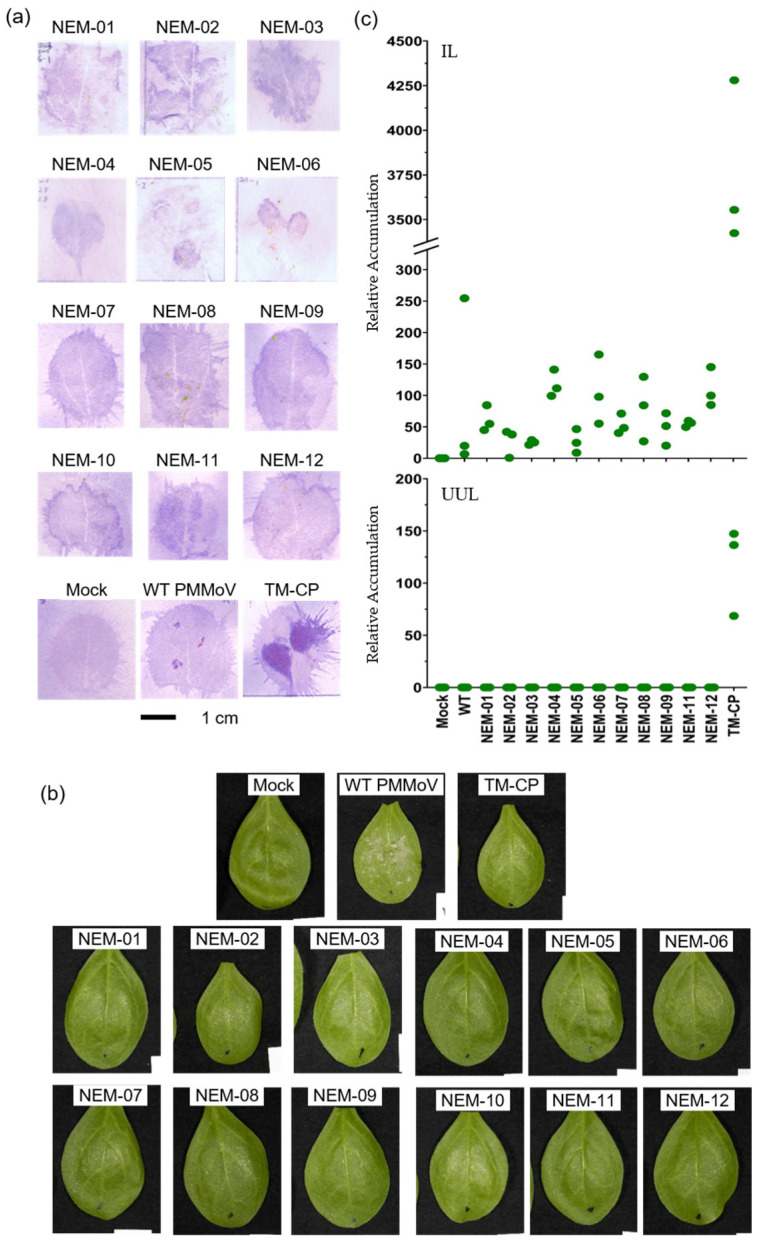
Identification of NEMs. (**a**) *N. sylvestris* leaves were toothpick-inoculated with *Agrobacterium* clones harboring PMMoV with a mutated CP gene. The inoculated leaves were observed at 7 days post-inoculation (dpi), and those without necrotic spots were analyzed using a hammer blot immunoassay. The 1 cm bar applies to all photos in (**a**,**b**). (**b**) *N. sylvestris* leaves were surface-inoculated with the same *Agrobacterium* clones using carborundum and photographed at 7 dpi. (**c**) RNA was extracted from the surface-inoculated leaves (IL) and uninoculated upper leaves (UUL) of *N. sylvestris* at 9 dpi and 20 dpi, respectively. PMMoV spread was evaluated using quantitative RT-PCR.

We previously established a system to introduce random mutations in the PMMoV CP and evaluate the mutant CPs in a viral context [23]. In the system, the PMMoV (strain Iw; P_1,2_ pathotype) CP gene was randomly mutagenized by error-prone PCR, and the mutated fragments were introduced into a PMMoV construct for agroinfection. PMMoV constructs with mutated CP genes were inoculated using a toothpick onto *Nicotiana sylvestris* containing the *N*′ tobamovirus resistance gene. Visual examination of necrotic spots and virus detection by a hammer blot immunoassay identified 12 mutant candidates, NEM-01 to NEM-12, after *N*′ resistance-escaping mutants (NEMs) (Figure 1a). The resistance-escaping phenotype of the 12 candidates was confirmed by surface inoculation of agroinfection clones (Figure 1b). Sequencing of the entire CP gene revealed that NEM-2 and -10 were identical (Supplementary Figure S1); therefore, we further analyzed the 11 candidates, excluding NEM-10. The NEMs had two to six amino acid changes from the wild-type CP (Supplementary Figure S2). They replicated slightly more than the wild-type PMMoV (WT) to different extents within the NEMs, but much less than PMMoV with TMV-CP (TM-CP) in surface-inoculated *N. sylvestris* leaves (Figure 1c, upper panel). Consistent with the low accumulation in the inoculated leaves, the mutants failed to infect the uninoculated upper leaves, unlike TM-CP (Figure 1c, lower panel). These results suggest that the NEMs escaped but did not break *N*′-mediated resistance in *N. sylvestris*. Additionally, none of the NEMs induced delayed and diffuse cell death (Figure 1b), which was induced by an *L*^3^-breaking variant in *L*^3^-resistant plants [6], suggesting that they did not activate the effector-triggered immunity (ETI) response [24,25] in *N. sylvestris*.

### 2.2. Minimal Mutations for Escaping N′-Mediated Resistance Reduce Virus Accumulation and Virulence in Pepper Plants

Because the NEMs showed reduced virus accumulation and failed to infect *N. sylvestris* systemically, we identified the minimal mutations required for escaping *N*′-mediated resistance by separating NEMs’ mutations using chimera construction and site-directed mutagenesis. NEMs and their derivatives were inoculated onto *N. sylvestris* using toothpicks to evaluate resistance escape. They were also agroinfiltrated into *N. benthamiana* leaves to evaluate the virus accumulation by a hammer blot immunoassay. During the optimization of *N. benthamiana* agroinfiltration, we found that agroinfiltration with a high bacterial density resulted in reduced NEM accumulation in the infiltrated areas. Therefore, we reduced the bacterial density to 0.01 OD unit/mL for evaluation (Supplementary Figure S3).

**Figure 2:**
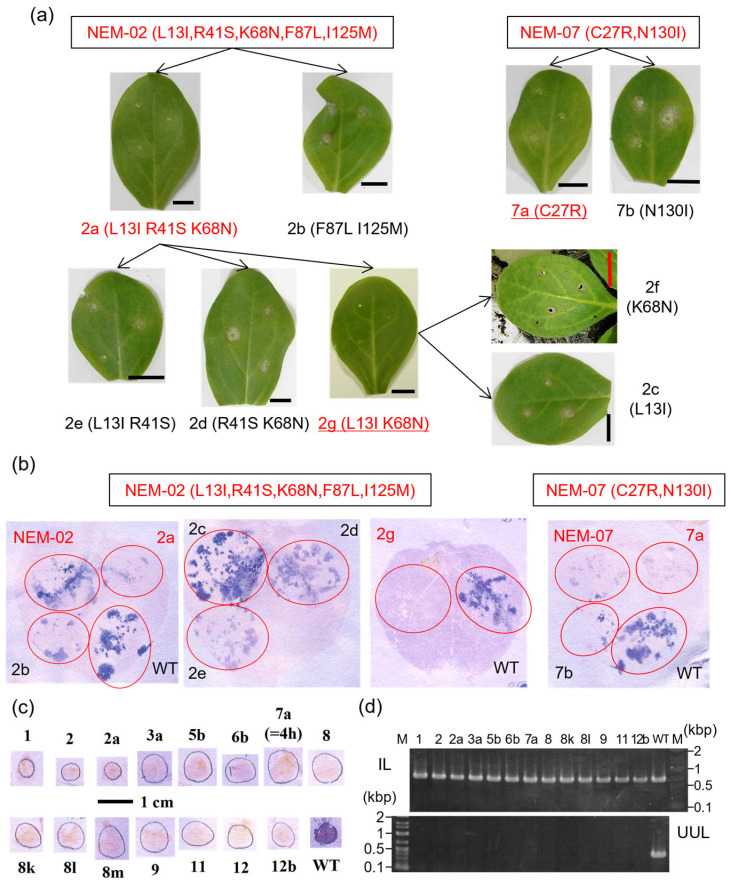
Identification of minimally required mutations in NEMs and their effects on pepper pathogenesis. (**a**) Evaluation of the *N*′-escaping phenotype. *N. sylvestris* leaves were toothpick-inoculated with *Agrobacterium* clones harboring PMMoV NEM derivative constructs and photographed at 7 dpi. The representative results for NEM-02 and -07 are shown. Derivatives were named with the NEM’s number plus an alphabet. The entire list of NEMs’ derivatives is presented in Supplementary Table S1. The bars in each panel denote 1 cm. (**b**) Evaluation of virus accumulation. Wild type (WT), NEMs, and their derivatives were agroinfiltrated into *N. benthamiana* leaves, and virus accumulation was evaluated using a hammer blot immunoassay. NEMs and their derivatives that escaped *N*′-mediated resistance are indicated in red. (**c**,**d**) Selected NEMs and derivatives were inoculated onto a PMMoV-susceptible pepper cultivar (*Capsicum annuum* cv. Shosuke) and analyzed by stem blot (**c**) and RT-PCR (**d**) of RNA from inoculated (IL) and uninoculated upper leaves (UUL).

We narrowed down the mutations step-by-step as follows: first, mutations in the N- and C-terminal halves were separated; second, when the N-terminal half contained the escaping mutations, those in the first and second quarters were separated, while for those in the C-terminal half, mutations in the third and fourth quarters were separated; when neither half did, each mutation was introduced separately or in combination. When we found resistance escape in a combination of two mutations among three candidates, the other (for example, R41S in NEM-02; Figure 2a) was not examined independently.

The results are summarized in Supplementary Table S1, and two representative results are shown (Figure 2a,b). In NEM-02, two amino acid substitutions were required to escape *N*′-mediated resistance, and the virus with the mutations accumulated less than the parental NEM-02. In NEM-07, a single mutation conditioned the *N*′-escaping capacity and reduced virus accumulation. Some derivatives, such as NEM-02 derivative 2 g, showed further reduced accumulation. In contrast, other derivatives showed a slightly milder reduction in virus accumulation (Supplementary Figure S3c, derivative 3a).

Hypothesizing that the NEMs’ derivatives that showed slightly more accumulation than the parental NEMs could systemically infect pepper plants, which are the natural host of PMMoV, we tested the infectivity of selected NEMs and derivatives in *Capsicum annuum* cv. Shosuke (+/+), which lacks the *L* resistance gene. However, this was not the case. The surface-inoculated plants supported virus replication in the inoculated leaves (Figure 2d and Supplementary Figure S4), but not systemic infection. We failed to detect viruses in the stems above the inoculated and uninoculated upper leaves, except for the WT (Figure 2c,d and Supplementary Figure S4). These results suggest that the amino acid mutations required for escaping *N*′-mediated resistance compromise PMMoV’s systemic infectivity.

### 2.3. Reduced Accumulation Facilitates Escape from Different Resistance Genes

A reduction in the accumulation of viruses accompanied the escape from *N*′-mediated resistance in NEMs. These findings raise the question of whether the lowered accumulation or the structural changes condition the escaping phenotype. We previously reported that the mode of recognition by the *N*′ protein differs from that of the allelic pepper L proteins [19]. Therefore, we tested whether NEMs could escape recognition by the L^3^ and L^4^ proteins. Both resistance proteins recognize a wide range of tobamovirus CPs, including those of PMMoV strains, except for those from resistance breakers [7,11,12,13,14,15,16].

**Figure 3:**
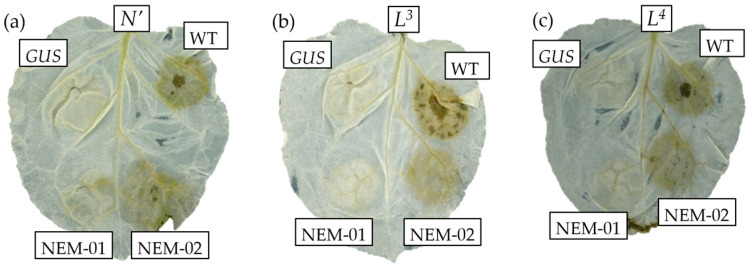
Evaluation of NEMs in the activation of *L*^3^- and *L*^4^-mediated resistance responses. *N. benthamiana* leaves were agroinfiltrated with wild-type (WT), *GUS*-expressing (control), and NEM constructs in combination with expression constructs of *N*′ (**a**), *L*^3^ (**b**), and *L*^4^ (**c**). Leaves were harvested 9 days post-infiltration, decolorized with ethanol, and photographed.

The expression constructs for N′, L^3^, and L^4^ proteins were transiently co-expressed with WT or NEMs in *N. benthamiana* leaves via agroinfiltration. A *GUS*-expressing construct served as a control. Unexpectedly, NEM-02 induced a hypersensitive response (HR), albeit to a lesser extent, when co-expressed with N′ (Figure 3a). It also induced HR with the L^3^ and L^4^ proteins (Figure 3b,c). Because NEM-02 has never induced a resistance response in the inoculated *N. sylvestris* leaves, the induction of HR in the co-infiltrated leaves would depend on high expression levels of the N′ protein. Other NEMs did not induce HR as expected with N′ or with L^3^ or L^4^ (Supplementary Figure S5). These results suggest that the reduced accumulation of Avr proteins may cause escape from resistance.

### 2.4. Systemic Infection of N. Benthamiana with NEMs with Attenuated Virulence

To test the lack of systemic infectivity in NEMs, we tested them in *N. benthamiana*, which is known to be highly susceptible to different virus species. Initial attempts using agroinfiltration resulted in poor systemic infectivity (Supplementary Figure S6).

**Figure 4:**
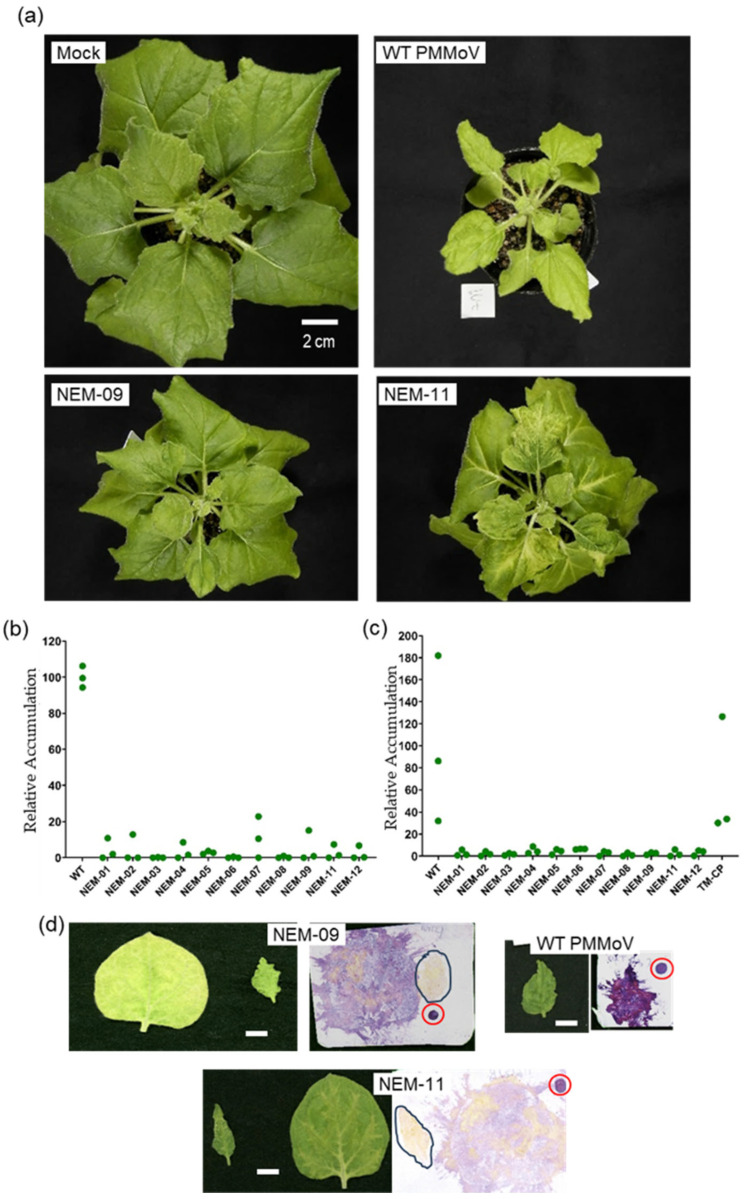
Systemic infection of *N. benthamiana* with NEMs. (**a**) Mock-inoculated plant (Mock) and those infected with wild-type (WT) and representative NEMs. The plants were photographed 3 weeks post-inoculation. The bar applies to all four panels. Plants were evaluated for relative virus accumulation in uninoculated upper leaves using ELISA (**b**) and RT-qPCR (**c**). In (**c**), plants infected with PMMoV expressing TMV CP (TM-CP) were included. (**d**) Age-dependent accumulation of NEMs. Young (small leaves; outlined in blots) and old uninoculated leaves were harvested from systemically infected plants 5 weeks post-inoculation and evaluated for virus accumulation using a hammer blot immunoassay. The results for the WT and two representative NEMs are shown. Positive controls for normalizing the color development are highlighted with red circles.

However, surface inoculation onto young plants consistently supported NEMs’ systemic infection (Figure 4a and Supplementary Figure S7). After surface inoculation of 3-week-old plants with NEMs’ *Agrobacterium* clones, all plants showed stunt and deformation of leaves in 3 weeks (Figure 4a and Supplementary Figure S7a). Some plants, including those infected with NEM-11, exhibited mosaic symptoms (Figure 4a). However, leaf deformation and stunt symptoms were much milder than those in plants infected with WT and TM-CP viruses, indicating that NEMs exhibit attenuated virulence. Infection of 2-week-old plants resulted in severe stunting but highlighted the attenuated virulence compared to WT and TM-CP viruses (Supplementary Figure S7b). These results suggest that NEMs retain the capacity to function as CP required for systemic spread.

The systemic spread of NEMs was evaluated using ELISA and RT-qPCR, revealing that their accumulation was severely compromised in uninoculated upper leaves (Figure 4b,c). We tested young leaves in these assays because younger leaves are generally more active and contain more viruses. However, we found that NEMs exhibited age-dependent accumulation: they were barely detectable in newly emerging leaves but were detectable in old, senescent leaves (Figure 4d and Supplementary Figure S8).

**Figure 5:**
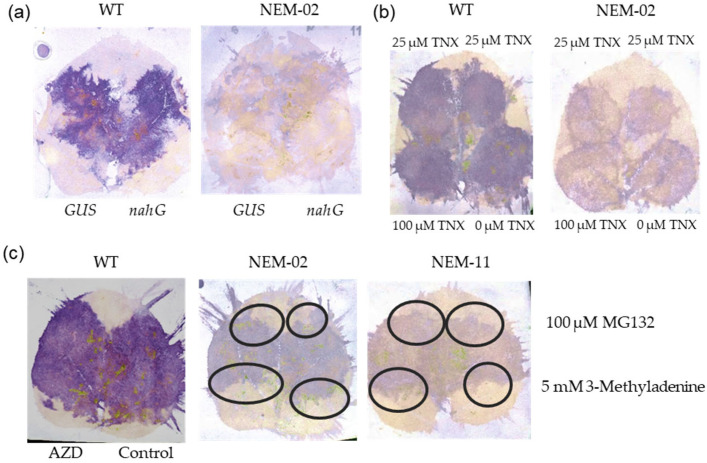
Search for the cause of low accumulation of NEMs in agroinfiltrated *N. benthamiana*. Virus accumulation was evaluated using a hammer blot immunoassay. (**a**) Effect of *nahG* expression on virus accumulation. A construct expressing the *GUS* gene was used as a control. The agrobacterium clones were infiltrated 2 days before agroinfiltration with wild-type (WT) PMMoV or NEM-02. (**b**) Effect of TNX on virus accumulation. The indicated concentrations of TNX were added to bacterial suspensions for WT and NEM-02 agroinfiltration. (**c**) Effect of proteasome inhibitor (MG132) and autophagy inhibitor (3-Methyladenine) on virus accumulation in NEMs. WT was co-agroinfiltrated with the autophagy inducer, AZD-8055. The areas infiltrated with the inhibitors are denoted by black circles.

Earlier experiments showed that NEMs accumulated less than WT in the infiltrated areas when agroinfiltrated with a high density of bacteria (Supplementary Figure S3b). Because agroinfiltration induces the expression of *PR1a*, a well-known defense marker gene, in tobacco [26], salicylic acid (SA)-mediated defense could be a cause for the reduced NEM accumulation. A salicylate hydroxylase gene, *nahG*, was transiently expressed via agroinfiltration before agroinfiltration-mediated NEM infection. However, no enhanced NEM accumulation was observed (Figure 5a and Supplementary Figure S9). We also co-infiltrated tenoxicam (TNX), an SA signaling inhibitor, with NEMs but did not observe any enhancement (Figure 5b and Supplementary Figure S10). Therefore, the reduced accumulation of NEMs cannot be attributed to SA-mediated defense.

Next, we hypothesized that an age-dependent loss of certain proteolytic activities, such as proteasome or autophagy, would facilitate the age-dependent accumulation of NEMs. Proteasome or autophagy inhibitors were infiltrated into the agroinfiltrated areas 3 days later, and the accumulation of NEMs was evaluated over the next 12 h (Figure 5c). A potent plant proteasome inhibitor, 100 μM MG132 [27], did not affect NEMs’ accumulation. Although compromised autophagy reportedly enhances PMMoV pathogenesis [28], preactivation of autophagy by AZD-8055 [29] did not affect the accumulation of WT and TM-CP, suggesting that autophagy does not directly break down these viruses (Figure 5c). Inhibition of autophagy by 5 mM 3-Methyladenine [30] did not affect NEMs’ accumulation (Figure 5c and Supplementary Figure S10). These results suggest that the known defense and degradation pathways tested in this study are unlikely to account for the reduced and age-dependent accumulation of NEMs, which should be confirmed using different methods, including the knockdown or knockout of key factors. Further investigation is necessary to examine the potential involvement of other factors, such as RNA instability, compromised CP translation, or alternative protein degradation pathways.

## 3. Discussion

### 3.1. Risk of N′-Mediated Resistance Breakage by PMMoV

The control of plant virus diseases largely relies on the use of resistant cultivars. Therefore, the emergence of resistance-breaking viruses is a serious problem in agriculture. A previous study pointed out that four major parameters correlate with the durability of plant virus resistance: the number and nature (transition or transversion) of amino acid substitutions in relevant virus factors required for the resistance breakdown, mutation rates, and fitness cost of the mutation in the virus life cycle [31]. Another study emphasized the correlation between resistance durability and evolutionary constraints in Avr factors, which correlate with the fitness penalty of resistance-breaking mutations [20].

The mutations in the NEMs are summarized in Table 1. Most of them involved transversions in the resistance-escaping phenotypes, except for the F35S mutation in NEM-03 and the C27R mutation in NEM-07 and NEM-04, which conferred resistance-escaping ability (Supplementary Table S1) with a single transition. Notably, none of the NEMs induced delayed or diffuse cell death in the inoculated *N. sylvestris* leaves (Figure 1b). These results suggest that NEMs do not activate the ETI response and are therefore likely to interact with the host in a compatible manner. In contrast, all the NEMs and derivatives tested were defective in systemic infection in *N. sylvestris* and pepper plants, indicating that *N*′-escaping mutations impose a severe fitness penalty on the viruses. Unlike PMMoV variants that overcame *L*^3^- and *L*^4^-mediated resistance, NEMs could not infect the susceptible pepper cultivar, distinguishing NEMs from *L*-breaking PMMoV strains. NEMs can also escape L^3^- and L^4^-mediated resistance. Therefore, it is formally possible that they break *L*-mediated resistance, although this is unlikely because of their inability to infect susceptible pepper plants systemically. Although NEMs are most unlikely to break down the *N*′-mediated resistance, it does not mean that the *N*′-mediated resistance is durable against PMMoV because the screening for PMMoV mutants in this study is unlikely to be saturated. To confirm the durability of *N*′-mediated resistance to PMMoV, further screening is needed, which can follow the procedures described herein.

When the durability of *N*′-mediated resistance to PMMoV is established in *N. sylvestris* and transgenic pepper plants expressing the *N*′ gene are generated, they should be subjected to long-term field or greenhouse trials under commercial production conditions to elucidate the effectiveness of the control measure and the practical risk of resistance breakdown. In light of the current knowledge on *N*′-escaping variants of PMMoV from this study, they are unlikely to cause epidemics because of their inability to cause systemic infection in pepper. However, further evolution of the variants could lead to the emergence of a so-called super strain, which could cause epidemics or even pandemics. Long-term trials could address this concern. Field or greenhouse trials also facilitate addressing another issue related to the transmission route: PMMoV is known to be soil-borne [32]. The trials will examine whether NEMs escape N′-mediated resistance and are unable to infect pepper plants systemically after invading the roots.

### 3.2. Accumulation of Avr Protein and Recognition by R Proteins

Studies have demonstrated various pathogen recognition by R proteins. Although LRR domains determine recognition specificities, as exemplified by allelic L proteins [6], the C-terminal domains of R proteins, including the CC of N′ and L proteins, and the third proteins have been shown to participate in the molecular interactions leading to pathogen perception [33]. Diverse viral proteins are recognized as Avr factors by cognate R proteins, and their accumulation in infected cells also varies. Therefore, the accumulation levels of Avr proteins might affect the recognition event differently, depending on the combination of R and Avr proteins.

Co-expression analysis with L^3^ and L^4^ proteins revealed that the responses to NEMs by *N*′, *L*^3^, and *L*^4^ resistance genes did not differ significantly (Figure 3). The results suggest that NEMs escape not only *N*′-mediated resistance but also *L*^3^- and *L*^4^-mediated resistance and that the reduced accumulation of Avr protein promotes resistance escape. In contrast, the NEM-02 derivatives 2b, 2d, and 2e accumulated less than the parental NEM-02 in *N. benthamiana* but activated *N. sylvestris* resistance responses (Figure 2a,b), suggesting that reduced accumulation alone is not sufficient for resistance escape. The results contradict the notion above. Different NEMs exhibited different virulence in *N. benthamiana*, suggesting that host cells perceive NEMs’ CPs. Therefore, the activation of the resistance response may require a longer protein–protein interaction than pathogenic interactions. Precise quantification of NEMs’ CPs and their dynamic analysis, which remain unestablished, would clarify whether reduced levels of Avr proteins or their structural changes are critical for escaping recognition by resistance proteins and elucidate the differences in protein–protein interactions between pathogenic and immune interactions.

### 3.3. Roles of Tobamovirus Coat Protein in Pathogenesis

Tobamovirus CP plays a role in long-distance movement but is dispensable for cell-to-cell movement. One study demonstrated that virion formation is necessary for long-distance movement [34], whereas another study claimed that CP activates DELLA-dependent suppression of SA pathways, which facilitates the systemic movement of CP-defective TMV in *N. benthamiana* [35]. In contrast, a study showed that TMV vector-mediated expression of CPs from PMMoV or cucumber green mottle mosaic virus activated SA pathways and induced severe symptoms in *Nicotiana tabacum* compared to TMV expressing its own CP [36]. Therefore, the involvement of the SA pathway in the long-distance movement of tobamoviruses remains controversial. All 11 NEMs consistently infected *N. benthamiana* systemically, suggesting their capability for long-distance movement.

All NEMs reached the upper uninoculated leaves and induced growth suppression in the infected plants. However, they exhibited highly attenuated virulence, as manifested by significantly milder growth suppression compared to WT and TM-CP (Figure 4 and Supplementary Figure S6). The mild growth suppression by MENs’ infection and the previous report of CP-mediated negative modulation of the SA pathway [35] led us to hypothesize that NEMs’ CPs interact with DELLA to a lesser extent and thus fail to evade SA-mediated defense, which was disproven as described (Figure 5a,b). This attenuation correlated with reduced virus accumulation, which could result from RNA silencing-mediated host defense. The tobamovirus replicase protein also acts as a silencing suppressor [37,38,39]. Although WT and NEMs share the wild-type replicase gene, their silencing suppressor functions can be modulated differently by the CPs of WT and NEMs. Further studies, including those on the involvement of RNA silencing, are needed to identify the cause of the attenuation of NEMs’ virulence.

Additionally, NEMs exhibited different mosaic patterns: NEM-09 induced ambiguous mosaic patterns similar to those of WT, whereas NEM-11 induced clear mosaic patterns (Figure 4a). They did not show significant differences in virus and viral RNA accumulation in systemically infected leaves (Figure 4b,c). Therefore, the difference in symptom types is unlikely to result from different virus accumulation levels. These results suggest that PMMoV CP plays a modulatory role in symptom development. The mechanism underlying symptom modulation remains unclear. A possible role of CP in symptom modulation could involve interactions with the replicase, as mosaic patterns are reportedly determined by a race between virus spread and the spread of antiviral silencing [40]. Further analysis of CP-replicase interactions would provide additional insight into the mechanisms underlying the development of plant virus disease symptoms.

## 4. Materials and Methods

### 4.1. Construction of Viral Clones, Mutant Library, and Expression Vectors

The agroinfection construct of the wild-type (WT) PMMoV Iw strain, pBTPIW, has been previously described [6]. The library of PMMoV CP mutants was constructed as previously described [23]. The expression constructs for the *N*′, *L*^3^, and *L*^4^ genes have been previously reported [6,19]. The *GUS* expression vector has been previously described [41]. The *nahG* expression vector was constructed by replacing the *GUS* coding sequence with the PCR-amplified *nahG* coding sequence using pRI_Xb_A65nahG-F and nahG-pRI_SacIR primers (Supplementary Table S1) and PrimeSTAR GXL DNA polymerase (Takara Bio, Kusatsu, Japan).

### 4.2. Plant Growth

Plants were grown at 25 °C under a 16 h day and 8 h night cycle. For NEM screening, one-week-old *N. sylvestris* seedlings were transferred to Jiffy-7 (4.2 cm in diameter before swelling; Jiffy Products International, Kristiansand, Norway) and grown for two weeks. *N. sylvestris*, *N. benthamiana*, and pepper (*Capsicum annuum* cv. Shosuke) seedlings were transferred to pots (9 cm in diameter) containing a commercial soil mixture (Sakata Seed Co., Yokohama, Japan) and grown as described above.

### 4.3. Virus Inoculation and Agroinfiltration

*A. tumefaciens* harboring the agroinfection constructs were grown on selective plates for about 24 h. For tooth-pick inoculation, bacterial cells were collected using a toothpick or a plastic inoculation loop, suspended in a small amount of inoculation buffer (10 mM MES, pH 5.5, 10 mM MgCl_2_) containing 30 μM acetosyringone, and then kept for a few hours at room temperature. *N. sylvestris* seedlings were inoculated by pricking the leaves with a toothpick dipped in the bacterial suspension at the two-leaf stage. For surface inoculation, the leaves were dusted with carborundum and rubbed with 20 μL of *Agrobacterium* suspension in the inoculation buffer at a bacterial density of 1 optical density (OD) unit/mL using a plastic inoculation loop or gloved finger. *N. benthamiana* was agroinfiltrated as described previously [6] at the bacterial density indicated in the figure legends.

### 4.4. Hammer Blot Immunoassay

Tissue blots of leaves were prepared as described previously [42], and those of infected pepper stems were prepared by pressing the cut surface onto a sheet of filter paper. Immunostaining was performed using anti-PMMoV antiserum or anti-TMV serum (Japan Plant Protection Association, Ushiku, Japan) as the primary antibodies and goat anti-rabbit IgG alkaline phosphatase conjugate (BioRad, Hercules, CA, USA) as the secondary antibody. Nitro blue tetrazolium and 5-bromo-4-chloro-3-indolyl-phosphate (Nakalai Tesque, Kyoto, Japan) were used as substrates for chromogenic reaction.

### 4.5. Detection of HR

HR induced by the co-expression of resistance genes and viral constructs was detected by visual observation after decolorizing the chlorophyll pigments using ethanol. Leaves were harvested 9 days post-infiltration and immersed in 99% ethanol overnight, followed by treatment with 70% ethanol for 2 days to enhance lesion visualization [6].

### 4.6. Virus Quantification by ELISA

*N. benthamiana* leaves infected with WT and NEMs were homogenized in 10 mL/g of phosphate-buffered saline containing 0.05% Tween-20 and 2% Polyvinyl pyrrolidone (Nakalai Tesque). Viruses were quantified using the PMMoV DAS-ELISA kit (Japan Plant Protection Association) and a colorimetric assay using a p-nitrophenyl phosphate substrate (Nakalai Tesque). Absorbance at 405 nm (A_405_) was determined using a microplate reader. The relative virus quantity in the NEM samples was calculated as a percentage of the average A_405_ of the WT sample after subtracting the A_405_ of the uninfected leaf.

### 4.7. Detection and Quantification of Viral RNA

RNA was extracted and reverse transcribed as described previously [43]. PMMoV RNA was detected using the primers PMF1 and PMR1, as described (Supplementary Table S2). Quantitative PCR was performed using KAPA SYBR Fast qPCR (Kapa Biosystems, Wilmington, MA, USA) with PMMoVqP02F and PMMoVqP02R for PMMoV (a part of the movement protein-coding sequence that WT, TM-CP, and NEMs share) and Nico18SqP-F and Nicol18qP-R for 18S ribosomal RNA as the internal reference (Supplementary Table S2). The relative amounts to the WT (average of three biological replicates) were calculated using the delta-delta-*C^T^* method.

### 4.8. Inhibitors and Autophagy Inducer Administration

TNX (Tokyo Chemical Industry, Tokyo, Japan) was dissolved in dimethyl sulfoxide (DMSO; Nacalai Tesque). The 100 mM stock solution was added to the inoculation buffer to achieve final concentrations of 200 or 50 μM, and the diluted reagents were mixed with equal volumes of bacterial suspensions. Aqueous solutions of 100 μM MG132 (FUJIFILM Wako, Osaka, Japan) or 5 mM 3-Methyladenine (Tokyo Chemical Industry) were infiltrated into leaves that were agroinfiltrated 3 days before with viral constructs so that the infiltrated areas partially overlapped with the agroinfiltrated areas. The autophagy inducer AZD-8055 (MedChemExpress, Monmouth Junction, NJ, USA) was dissolved in DMSO and added to the bacterial suspension to a final concentration of 1 μM, similarly to TNX.

## Figures and Tables

**Table 1:** List of NEMs and derivatives.

Clones ^a^	Amino Acid Substitutions ^b^	No. ^c^	Mode ^d^	Accum. ^e^	R-Escape ^f^	Systemic Infectivity		
						*Nsyl* ^g^	*Cann* ^h^	*Nben* ^i^
NEM-01	S5P L23I T54A P63S I94V N140S	6	S+V+S+V+S+S	+	*N*′, *L*^3^, *L*^4^	−	−	+
NEM-02	L13I R41S K68N F87L I125M	5	V+V+V+S+S	++	(*N*′), *L*^3^, *L*^4^	−	−	+
NEM-03	Y12C F35S Q45R N73S S148R	5	S+S+S+S+V	+	*N*′, *L*^3^, *L*^4^	−	−	+
NEM-04	N25D C27R Q36H V51A F70S M129K	6	S+S+V+S+S+V	+	*N*′, *L*^3^, *L*^4^	−	−	+
NEM-05	Y12S F62N	2	V+(V+V)	+	*N*′, *L*^3^, *L*^4^	−	−	+
NEM-06	T57S T103S R122G Y139D	4	V+V+S+V	+	*N*′, *L*^3^, *L*^4^	−	−	+
NEM-07	C27R N130I	2	S+V	+	*N*′, *L*^3^, *L*^4^	−	−	+
NEM-08	N101D L128H F144Y W152R	4	S+V+V+S	+	*N*′, *L*^3^, *L*^4^	−	−	+
NEM-09	N25H Q38R Q46P Q141H	4	V+S+V+V	+	*N*′, *L*^3^, *L*^4^	−	−	+
NEM-11	Y2N N8K S49P S78P V114A	5	V+V+S+S+S	++	*N*′, *L*^3^, *L*^4^	−	−	+
NEM-12	F35L R41G F62L F70S	4	S+S+S+S	++	*N*′, *L*^3^, *L*^4^	−	−	+

^a^ Names of mutant clones. ^b^ Amino acid substitutions in the mutants or derivatives as compared to wild-type PMMoV-Iw. ^c^ Number of amino acid substitutions in the mutants or derivatives. ^d^ Mode of nucleotide substitutions involved in the amino acid substitutions: S, transition; V, transversion. Two nucleotide substitutions involved in a single amino acid substitution are shown in brackets. ^e^ CP accumulation levels in agroinfiltrated *N. benthamiana* were determined using hammer blot immunodetection. ++, slightly lower than that in WT; +, significantly lower than that in WT. ^f^ Resistance genes escaped in *N. sylvestris* infection (*N*′) and co-expression analysis. NEM-02 was recognized by *N*′ in the co-expression analysis, although it escaped *N*′ in *N. sylvestris* and was therefore indicated in a bracket. ^g^ Systemic infection in *N. sylvestris*. −, undetected by either RT-qPCR or tissue blotting. ^h^ Systemic infection in *C. annuum* cv. Shosuke. −, undetected by either RT-PCR or tissue blotting. ^i^ Systemic infection in *N. benthamiana*. +, symptomatic infection with viruses detected by RT-qPCR, ELISA, and tissue blotting.

## Data Availability

The raw data supporting the conclusions of this study will be made available by the authors upon request. The data are not publicly available because all representative results are presented in the main or supplementary figures.

## Supplementary Materials

The following supporting information can be downloaded at https://www.mdpi.com/article/10.3390/plants14162471/s1, Supplementary Figure S1. Nucleotide sequence alignment of NEMs; Supplementary Figure S2. Amino acid sequence alignment of NEMs; Supplementary Figure S3. Hammer blot immunoassay of PMMoV CP; Supplementary Figure S4. Hammer blot immunoassay of the inoculated and uninoculated upper leaves of pepper (*Capsicum annuum* cv. Shosuke) plants infected with selected NEMs and their derivatives; Supplementary Figure S5. Hypersensitive reaction (HR) in *N. benthamiana* leaves co-expressed with resistance genes and NEMs; Supplementary Figure S6. Characterization of NEMs in agroinfiltrated *N. benthamiana;* Supplementary Figure S7. Systemic infection of *N. benthamiana* with NEMs; Supplementary Figure S8. Age-dependent accumulation of NEMs in systemically infected *N. benthamiana*; Supplementary Figure S9. Effect of salicylate hydroxylase gene expression on the accumulation of NEMs in agroinfiltrated *N. benthamiana* leaves; Supplementary Figure S10. Effect of salicylic acid signaling inhibitor on the accumulation of NEMs in agroinfiltrated *N. benthamiana* leaves; Supplementary Figure S11. Effect of proteasome or autophagy inhibitors on the accumulation of NEMs in agroinfiltrated *N. benthamiana* leaves; Supplementary Table S1. Summary of analyses of NEMs and their derivatives; Supplementary Table S2. Primers used in this study.
