# Supplementary material for: A Tradeoff Between the Escape from N′-Mediated Resistance and Virulence in Pepper Mild Mottle Virus Through Reduced Virus Accumulation
Source: Plants (Basel). 2025 Aug 9;14(16):2471. doi: 10.3390/plants14162471 (PMC12389172; doi:10.3390/plants14162471)
Supplement: Supplementary file 1 [file plants-14-02471-s001.zip › Supple-Figs-R1.pdf]

```
PMMoV-Iw   ATGGCTTACACAGTTTCCAGTGCCAATCAATTAGTGTATTTAGGTTCTGTATGGGCCGAT   60
NEM-01     ATGGCTTACACAGTTCCCAGTGCCAATCAATTAGTGTATTTAGGTTCTGTATGGGCCGAT   60
NEM-02     ATGGCTTACACAGTTTCCAGTGCCAATCAATTAGTGTATATAGGTTCTGTATGGGCCGAT   60
NEM-03     ATGGCTTACACAGTTTCCAGTGCCAATCAATTAGTGTGTTTAGGTTCTGTATGGGCCGAT   60
NEM-04     ATGGCTTACACAGTTTCCAGTGCCAATCAATTAGTTTATTTAGGTTCTGTATGGGCCGAT   60
NEM-05     ATGGCTTACACAGTTTCCAGTGCCAATCAATTAGTGTCTTTAGGTTCTGTATGGGCCGAT   60
NEM-06     ATGGCTTACACAGTTTCCAGTGCCAATCAATTAGTGTACTTAGGTTCTGTATGGGCCGAT   60
NEM-07     ATGGCTTACACAGTTTCCAGTGCCAATCAATTAGTGTATTTAGGTTCTGTATGGGCCGAT   60
NEM-08     ATGGCTTACACAGTTTCCAGTGCCAATCAATTAGTGTATTTAGGTTCTGTATGGGCCGAT   60
NEM-09     ATGGCTTACACAGTTTCCAGTGCCAATCAATTAGTGTACTTAGGTTCTGTATGGGCCGAT   60
NEM-10     ATGGCTTACACAGTTTCCAGTGCCAATCAATTAGTGTATATAGGTTCTGTATGGGCCGAT   60
NEM-11     ATGGCTAACACAGTTTCCAGTGCCAAACAATTAGTGTATTTAGGTTCTGTATGGGCCGAT   60
NEM-12     ATGGCTTACACAGTTTCCAGTGCCAATCAATTAGTGTATTTAGGTTCTGTATGGGCCGAT   60

PMMoV-Iw   CCATTAGAGTTACAAAATCTATGTACTTCGGCGTTAGGTAATCAGTTTCAAACACAGCAG   120
NEM-01     CCATTAGAGATACAAAATCTATGTACTTCGGCGTTAGGTAATCAGTTTCAAACACAGCAG   120
NEM-02     CCATTAGAGTTACAAAATCTATGTACTTCGGCGTTAGGTAATCAGTTTCAAACACAGCAG   120
NEM-03     CCATTAGAGTTACAAAATCTATGTACTTCGGCGTTAGGTAATCAGTCTCAAACACAGCAG   120
NEM-04     CCATTAGAGTTACAAGATCTACGTACTTCGGCGTTAGGTAATCAGTTTCATACACAGCAG   120
NEM-05     CCATTAGAGTTACAAAATCTATGTACTTCGGCGTTAGGTAATCAGTTTCAAACACAGCAG   120
NEM-06     CCATTAGAGTTACAAAATCTATGTACTTCGGCGTTAGGTAATCAGTTTCAAACACAGCAG   120
NEM-07     CCATTAGAGTTACAAAATCTACGTACTTCGGCGTTAGGTAATCAGTTTCAAACACAGCAG   120
NEM-08     CCATTAGAGTTACAAAATCTATGTACTTCGGCGTTAGGTAATCAGTTTCAAACACAGCAG   120
NEM-09     CCATTAGAGTTACAACATCTATGTACTTCGGCGTTAGGTAATCAGTTTCAAACACGGCAG   120
NEM-10     CCATTAGAGTTACAAAATCTATGTACTTCGGCGTTAGGTAATCAGTTTCAAACACAGCAG   120
NEM-11     CCATTAGAGTTACAAAATCTATGTACTTCGGCGTTAGGTAATCAGTTTCAAACACAGCAG   120
NEM-12     CCATTAGAGTTACAAAATCTATGTACTTCGGCGTTAGGTAATCAGCTTCAAACACAGCAG   120

-

PMMoV-Iw   GCTAGAACTACGGTTCAACAGCAGTTCTCTGATGTGTGGAAGACCATACCGACCGCTACA   180
NEM-01     GCTAGAACTACGGTTCAACAGCAGTTCTCTGATGTGTGGAAGGCCATACCGACCGCTACA   180
NEM-02     GCTAGTACTACGGTTCAACAGCAGTTCTCTGATGTGTGGAAGACCATACCGACCGCTACA   180
NEM-03     GCTAGAACTACGGTTCGACAGCAGTTCTCTGATGTGTGGAAGACCATACCGACCGCTACA   180
NEM-04     GCTAGAACTACGGTTCAACAGCAGTTCTCTGATGCGTGGAAGACCATTCCGACCGCTACA   180
NEM-05     GCTAGAACTACGGTACAACAGCAGTTCTCCGATGTGTGGAAGACCATACCGACCGCTACA   180
NEM-06     GCTAGAACTACGGTTCAACAGCAGTTCTCTGATGTGTGGAAGTCCATACCGACCGCTACA   180
NEM-07     GCTAGAACTACGGTTCAACAGCAGTTCTCTGATGTGTGGAAGACCATACCGACCGCTACA   180
NEM-08     GCTAGAACTACGGTTCAACAGCAGTTCTCTGATGTGTGGAAGACCATACCGACCGCTACA   180
NEM-09     GCTAGAACTACGGTTCAACCGCAGTTCTCTGATGTGTGGAAGACCATACCGACCGCTACA   180
NEM-10     GCTAGTACTACGGTTCAACAGCAGTTCTCTGATGTGTGGAAGACCATACCGACCGCTACA   180
NEM-11     GCTAGAACTACGGTTCAACAGCAGTTCCCTGATGTGTGGAAGACCATACCGACCGCTACA   180
NEM-12     GCTGGAACTACGGTTCAACAGCAGTTCTCTGATGTGTGGAAGACCATACCGACCGCTACA   180

PMMoV-Iw   GTTAGATTTCCTGCTACTGGTTTCAAAGTTTTTCGATATAATGCCGTGCTAGATTCTCTA   240
NEM-01     GTTAGATTTTCTGCTACTGGTTTCAAAGTTTTTCGATATAATGCCGTGCTAGATTCTCTA   240
NEM-02     GTTAGATTTCCAGCTACTGGTTTCAATGTTTTTCGATATAATGCCGTGCTAGATTCTCTA   240
NEM-03     GTTAGATTTCCTGCTACTGGTTTCAAAGTTTTTCGATATAGTGCCGTGCTAGATTCTCTC   240
NEM-04     GTTAGATTTCCTGCTACTGGTTTCAAAGTTTCTCGATATAATGCCGTGCTAGATTCTCTA   240
NEM-05     GTTAGAAATCCTGCTACTGGTTTCAAAGTTTTTCGATATAATGCCGTGCTAGATTCTCTA   240
NEM-06     GTTAGATTTCCTGCTACTGGTTTCAAAGTTTTTCGATATAATGCCGTGCTAGATTCTCTA   240
NEM-07     GTTAGATTTCCTGCTACTGGTTTCAAAGTTTTTCGATATAATGCCGTGCTGGATTCTCTA   240
NEM-08     GTTAGATTTCCTGCTACTGGTTTCAAAGTTTTTCGATATAATGCCGTGCTAGATTCTCTA   240
NEM-09     GTTAGATTTCCTGCTACTGGTTTCAAAGTTTTTCGATATAATGCCGTGCTAGATTCTCTA   240
NEM-10     GTTAGATTTCCAGCTACTGGTTTCAATGTTTTTCGATATAATGCCGTGCTAGATTCTCTA   240
NEM-11     GTTAGATTTCCTGCTACTGGTTTCAAAGTTTTTCGATATAATGCCGTGCTAGATCCTCTA   240
NEM-12     GTTAGACTTCCTGCTACTGGTTTCAAAGTTTCTCGATATAATGCCGTGCTAGATTCTCTA   240
```

Continues to the next page

Continued from the previous page

```
PMMoV-Iw   GTGTCGGCACTTCTCGGAGCCTTTGATACTCGGAATAGGATAATAGAAGTAGAAAATCCG   300
NEM-01     GTGTCGGCACTTCTCGGAGCCTTTGATACTCGGAATAGGATAGTAGAAGTAGAAAATCCG   300
NEM-02     GTGTCGGCACTTCTCGGAGCCTTAGATACTCGGAATAGGATAATAGAAGTAGAAAATCCG   300
NEM-03     GTGTCGGCACTTCTCGGAGCCTTTGATACTCGGAATAGGATAATAGAAGTAGAAAATCCG   300
NEM-04     GTGTCGGCACTTCTCGGAGCCTTTGATACTCGGAATAGGATAATAGAAGTAGAAAATCCG   300
NEM-05     GTGTCGGCACTTCTCGGAGCCTTTGATACTCGGAATAGGATAATAGAAGTAGAAAATCCG   300
NEM-06     GTGTCGGCACTTCTCGGAGCCTTTGATACTCGGAATAGGATAATAGAAGTAGAAAATCCG   300
NEM-07     GTGTCGGCACTTCTCGGAGCCTTTGATACTCGGAATAGGATAATAGAAGTAGAAAATCCG   300
NEM-08     GTGTCGGCACTTCTCGGAGCCTTTGATACTCGGAATAGGATAATAGAAGTAGAAAATCCG   300
NEM-09     GTGTCGGCACTTCTCGGAGCCTTTGATACTCGGAATAGGATAATAGAAGTAGAAAATCCG   300
NEM-10     GTGTCGGCACTTCTCGGAGCCTTAGATACTCGGAATAGGATAATAGAAGTAGAAAATCCG   300
NEM-11     GTGTCGGCACTTCTCGGAGCCTTTGATACTCGGAATAGGATAATAGAAGTAGAAAATCCG   300
NEM-12     GTGTCGGCACTTCTCGGAGCCTTTGATACTCGGAATAGGATAATAGAAGTAGAAAATCCG   300

PMMoV-Iw   CAAAATCCTACAACTGCCGAGACGCTTGATGCGACGAGGCGGGTAGATGATGCGACGGTG   360
NEM-01     CAAAATCCTACAACAGCCGAGACGCTTGATGCGACGAGGCGGGTAGATGATGCGACGGTG   360
NEM-02     CAAAATCCTACAACTGCCGAGACGCTTGATGCGACGAGGCGGGTAGATGATGCGACGGTG   360
NEM-03     CAAAATCCTACAACTGCCGAGACGCTTGATGCGACGAGGCGGGTAGATGATGCGACGGTG   360
NEM-04     CAAAATCCTACAACTGCCGAGACGCTTGATGCGACGAGGCGGGTAGATGATGCGACGGTG   360
NEM-05     CAAAATCCTACAACTGCCGAGACGCTTGATGCGACGAGGCGGGTAGATGATGCGACGGTG   360
NEM-06     CAAAATCCTTCAACTGCCGAGACGCTTGATGCGACGAGGCGGGTAGATGATGCGACGGTG   360
NEM-07     CAAAATCCTACAACTGCCGAGACGCTTGATGCGACGAGGCGGGTAGATGATGCGACAGTG   360
NEM-08     CAAGATCCTACAACTGCCGAGACGCTTGATGCGACGAGGCGGGTAGATGATGCGACGGTG   360
NEM-09     CAAAATCCTACAACTGCCGAGACGCTTGATGCGACGAGGCGGGTAGATGATGCGACGGTG   360
NEM-10     CAAAATCCTACAACTGCCGAGACGCTTGATGCGACGAGGCGGGTAGATGATGCGACGGTG   360
NEM-11     CAAAATCCTACAACTGCCGAGACGCTTGATGCGACGAGGCGGCAGATGATGCGACGGTG    360
NEM-12     CAAAATCCTACAACAGCCGAGACGCTTGATGCGACGAGGCGGGTAGATGATGCGACGGTG   360

PMMoV-Iw   GCCATTAGGGCCAGTATAAGTAACCTCATGAATGAGTTAGTTCGTGGCACGGGAATGTAC   420
NEM-01     GCCATTAGGGCCAGTATAAGTAACCTCATGAATGAGTTAGTTCGTGGCACGGGAATGTAC   420
NEM-02     GCCATTAGGGCCAGTATGAGTAACCTCATGAATGAGTTAGTTCGTGGCACGGGAATGTAC   420
NEM-03     GCCATTAGGGCCAGTATAAGTAACCTCATGAATGAGTTAGTTCGTGGCACGGGAATGTAC   420
NEM-04     GCCATTAGGGCCAGTATAAGTAACCTCAAGAATGAGTTAGTTCGTGGCACGGGAATGTAC   420
NEM-05     GCCATTAGGGCCAGTATAAGTAACCTCATGAATGAGTTAGTTCGTGGCACGGGAATGTAC   420
NEM-06     GCCATTGGGGCCAGTATAAGTAACCTCATGAATGAGTTAGTTCGTGGCACGGGAATGGAC   420
NEM-07     GCCATTAGGGCCAGTATAAGTAACCTCATGATTGAGTTAGTTCGTGGCACGGGAATGTAC   420
NEM-08     GCCATTAGGGCCAGTATAAGTAACCACATGAATGAGTTAGTTCGTGGCACGGGAATGTAC   420
NEM-09     GCCATTAGGGCCAGTATAAGCAACCTCATGAATGAGTTAGTTCGTGGCACGGGAATGTAC   420
NEM-10     GCCATTAGGGCCAGTATGAGTAACCTCATGAATGAGTTAGTTCGTGGCACGGGAATGTAC   420
NEM-11     GCCATTAGGGCCAGTATAAGTAACCTCATGAATGAGTTAGTTCGAGGCACGGGAATGTAC   420
NEM-12     GCCATTAGGGCCAGTATAAGTAACCTCATGAATGAGTTAGTTCGTGGCACGGGAATGTAC   420

PMMoV-Iw   AATCAAGCTCTGTTCGAGAGCGCGAGTGGACTCACCTGGGCTACAACTCCTTAA   474
NEM-01     AGTCAAGCTCTGTTCGAGAGCGCGAGTGGACTCACCTGGGCTACAACTCCTTAA   474
NEM-02     AATCAAGCTCTGTTCGAGAGCGCGAGTGGACTCACCTGGGCTACAACTCCTTAA   474
NEM-03     AATCAAGCTCTGTTCGAGAGCGCGAGAGGACTCACCTGGGCTACAACTCCTTAA   474
NEM-04     AATCAAGCTCTGTTCGAGAGCGCGAGTGGACTCACCTGGGCTACAACTCCTTAA   474
NEM-05     AATCAAGCTCTGTTCGAGAGCGCGAGTGGACTCACCTGGGCTACAACTCCTTAA   474
NEM-06     AATCAAGCTCTGTTCGAGAGCGCGAGCGGACTCACCTGGGCTACAACTCCTTAA   474
NEM-07     AATCAAGCTCTGTTCGAGAGCGCGAGTGGACTCACCTGGGCTACAACTCCTTAA   474
NEM-08     AATCAAGCTCTGTACGAGAGCGCGAGTGGACTCACCAGGGCCACAACTCCTTAA   474
NEM-09     AATCATGCTCTGTTCGAGAGCGCGAGTGGACTCACCTGGGCTACAACTCCTTAA   474
NEM-10     AATCAAGCTCTGTTCGAGAGCGCGAGTGGACTCACCTGGGCTACAACTCCTTAA   474
NEM-11     AATCAAGCTCTGTTCGAGAGCGCGAGTGGACTCACCTGGGCTACAACTCCTTAA   474
NEM-12     AATCAAGCTCTGTTCGAGAGCGCGAGTGGACTCACCTGGGCTACAACTCCTTAA   474
```

Supplementary Figure S1. Nucleotide sequence alignment of *N*´-escaping mutants (NEMs). The CP gene sequences of NEMs were determined and aligned using GENETYX software (GENETYX CORPORATION, Tokyo, Japan). Nucleotide substitutions are highlighted in red, and conserved sequences are boxed.

```
                   10        20        30        40        50        60        70        80
           ----:----|----:----|----:----|----:----|----:----|----:----|----:----|----:----|
PMMoV-Wt   MAYTVSSANQLVYLGSVWADPLELQNLCTSALGNQFQTQQARTTVQQQFSDVWKTIPTATVRFPATGFKVFRYNAVLDSL
NEM-01     .....P.................I..............................A........S................
NEM-02     .............I...........................S..........................N...........
NEM-03     ............C......................S.........R...........................S......
NEM-04     .........................D.R........H..............A..................S.........
NEM-05     ............S.................................................N.................
NEM-06     ......................................................S.........................
NEM-07     ...........................R....................................................
NEM-08     ................................................................................
NEM-09     .........................H............R.......P.................................
NEM-11     ..N.....K........................................P............................P.
NEM-12     ...................................L.....G....................L.......S.........

                   90       100       110       120       130       140       150     157
           ----:----|----:----|----:----|----:----|----:----|----:----|----:----|----:--
PMMoV-Iw   VSALLGAFDTRNRIIEVENPQNPTTAETLDATRRVDDATVAIRASISNLMNELVRGTGMYNQALFESASGLTWATTP
NEM-01     ..............V.............................................S................
NEM-02     .......L.....................................M...............................
NEM-03     ....................................................................R........
NEM-04    .................................................K...........................
NEM-05     .............................................................................
NEM-06     .......................S..................G................D.................
NEM-07     ..................................................I..........................
NEM-08     .....................D..........................H...............Y.......R....
NEM-09     .............................................................H...............
NEM-11     ..................................A..........................................
NEM-12     .............................................................................
```

Supplementary Figure S2. Amino acid sequence alignment of *N*´-escaping mutants (NEMs). The CP gene sequences of NEMs were determined and aligned using GENETYX software. Only the amino acid substituted are shown for the NEMs.

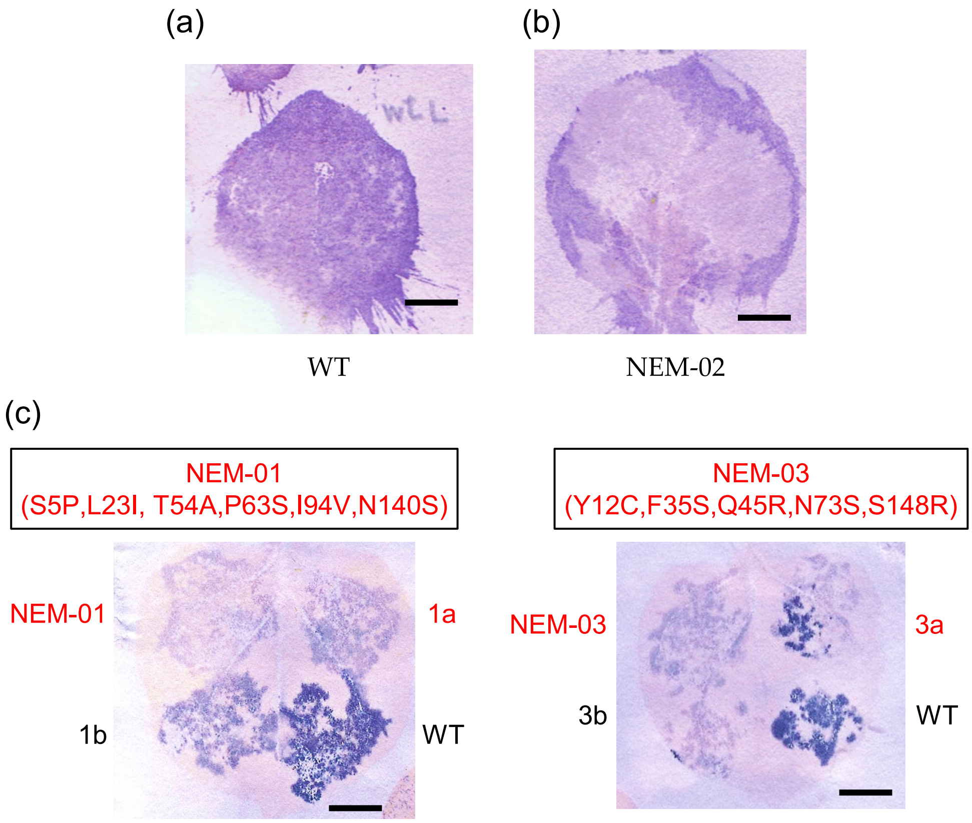

Supplementary Figure S3. Hammer blot immunoassay of PMMoV CP. *N. benthamiana* leaves were agroinfiltrated with wild-type (WT) (a) and *N*´-escaping mutants (NEMs), represented by NEM-02. (b) at a bacterial density of 1 OD unit/ml. (c) Representative results of the evaluation of NEMs' and their derivatives' accumulation levels. NEMs and their derivatives that escaped *N*´-mediated resistance are shown in red. All the results are summarized in Supplementary Table S1. Bar in each panel denotes 1 cm.

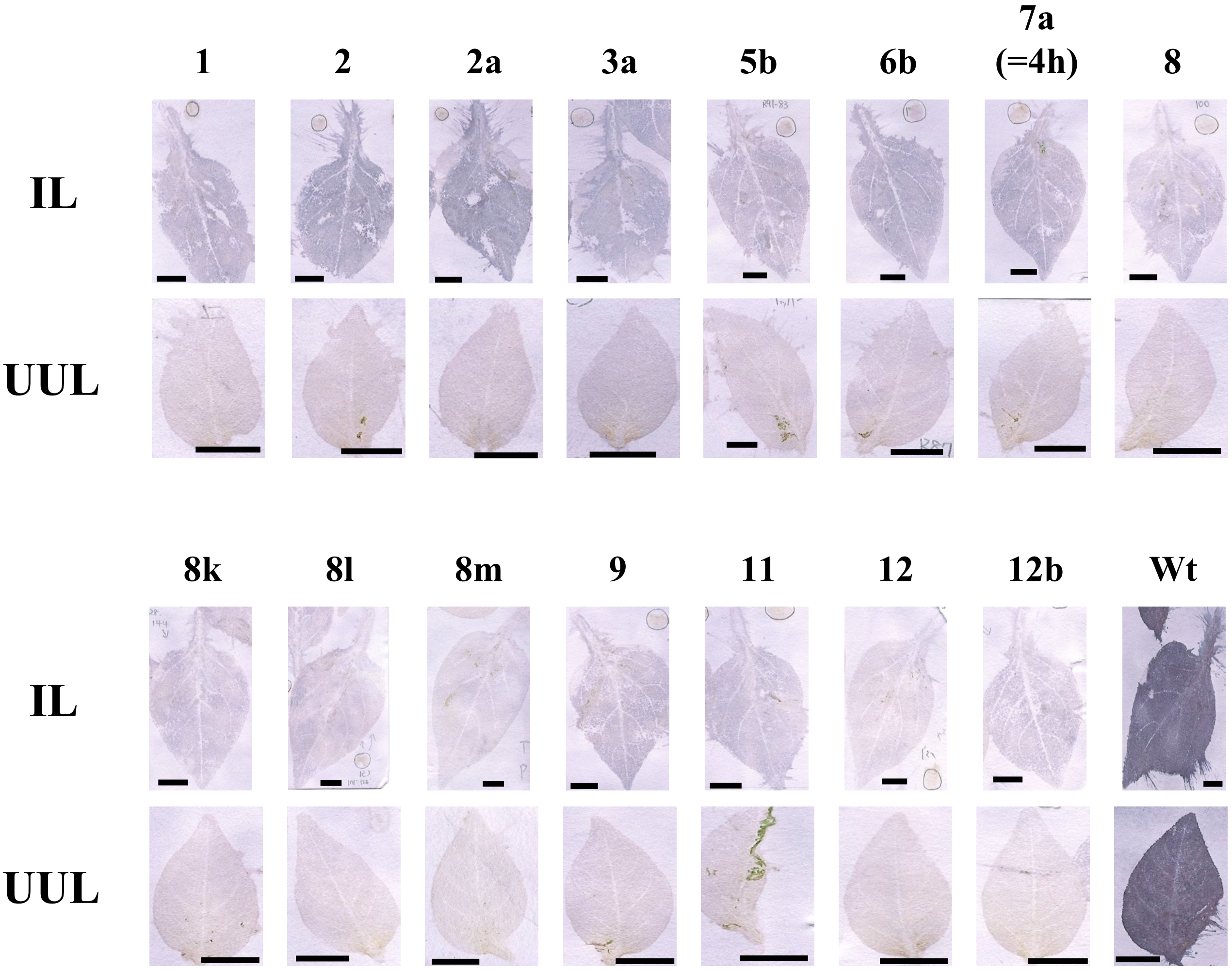

Supplementary Figure S4. Hammer blot immunoassay of the inoculated and uninoculated upper leaves of pepper (*Capsicum annuum* cv. Shosuke) plants infected with selected *N*´-escaping mutants (NEMs) and their derivatives. Inoculated (IL) and uninoculated upper (UUL) leaves of pepper plants surface-inoculated with *Agrobacterium* clones harboring selected viral clones were analyzed.

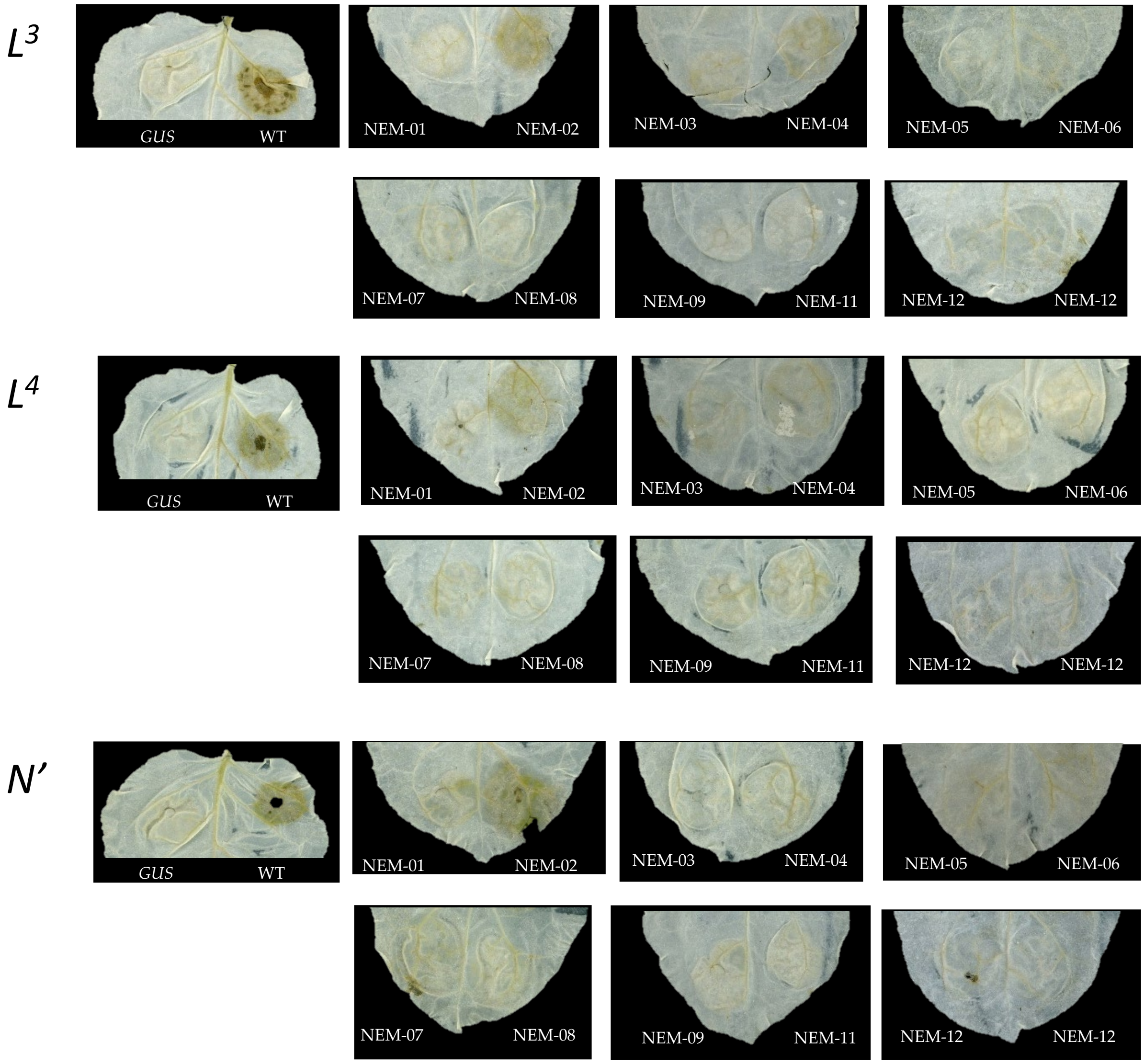

Supplementary Figure S5. Hypersensitive reaction (HR) in *N. benthamiana* leaves co-agroinfiltrated with resistance genes and *N*´-escaping mutants (NEMs). *N. benthamiana* leaves were co-agroinfiltrated with constructs for *N′*, L^3, or L^4 genes in combination with NEMs constructs. The infiltrated leaves were harvested 9 days post-infiltration, decolorized for visual enhancement of HR, and photographed.

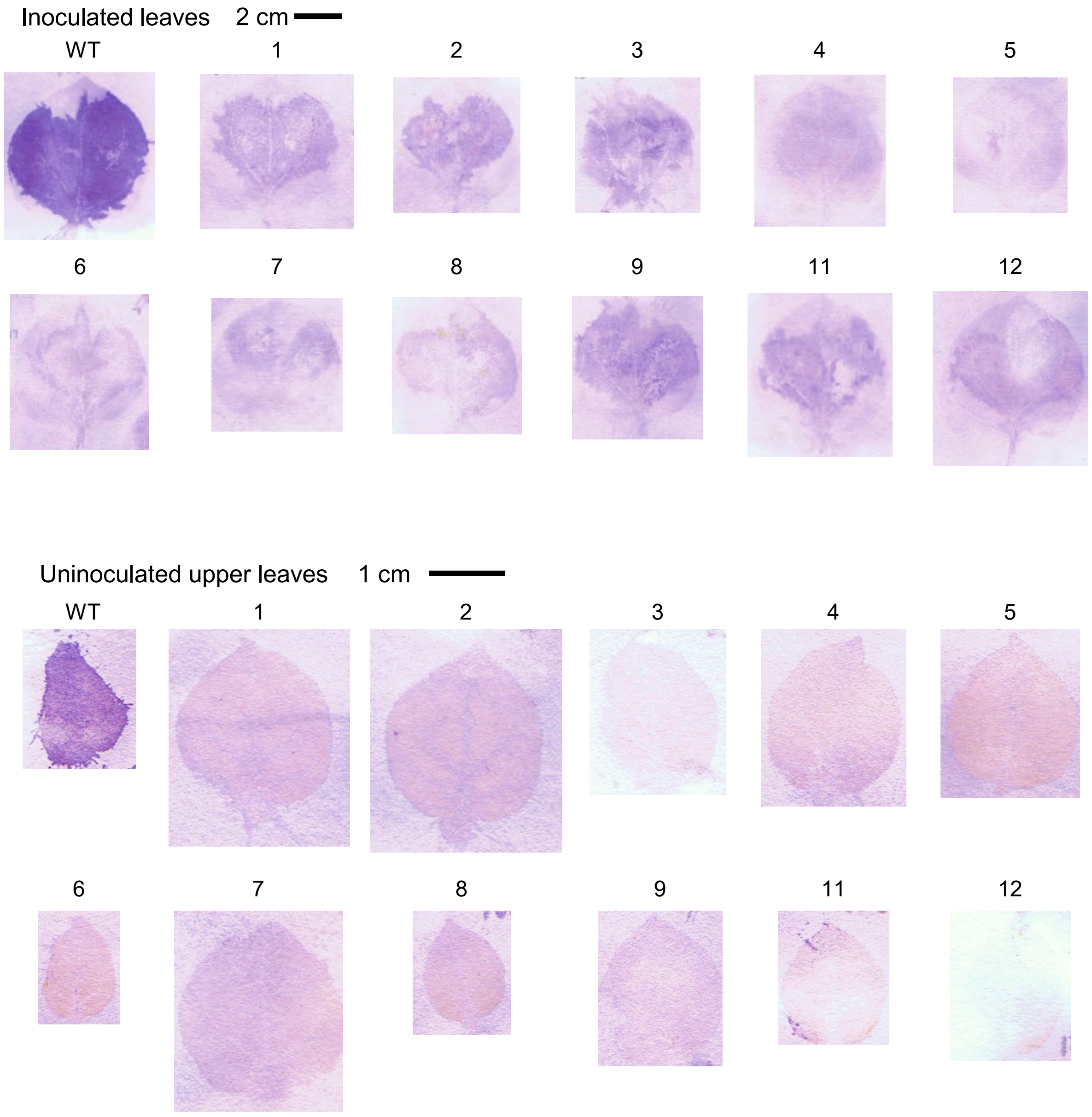

Supplementary Figure S6. Characterization of *N*´-escaping mutants (NEMs) in agroinfiltrated *N. benthamiana*. *N. benthamiana* leaves were agroinfiltrated with wild-type (WT) and NEMs PMMoV clones at a bacterial density of 0.1. Inoculated and uninoculated upper leaves were analyzed by hammer blot immunoassay at 7 dpi and 14 dpi, respectively. 1–12, NEM-01–12; Wt, wild-type PMMoV.

(a)

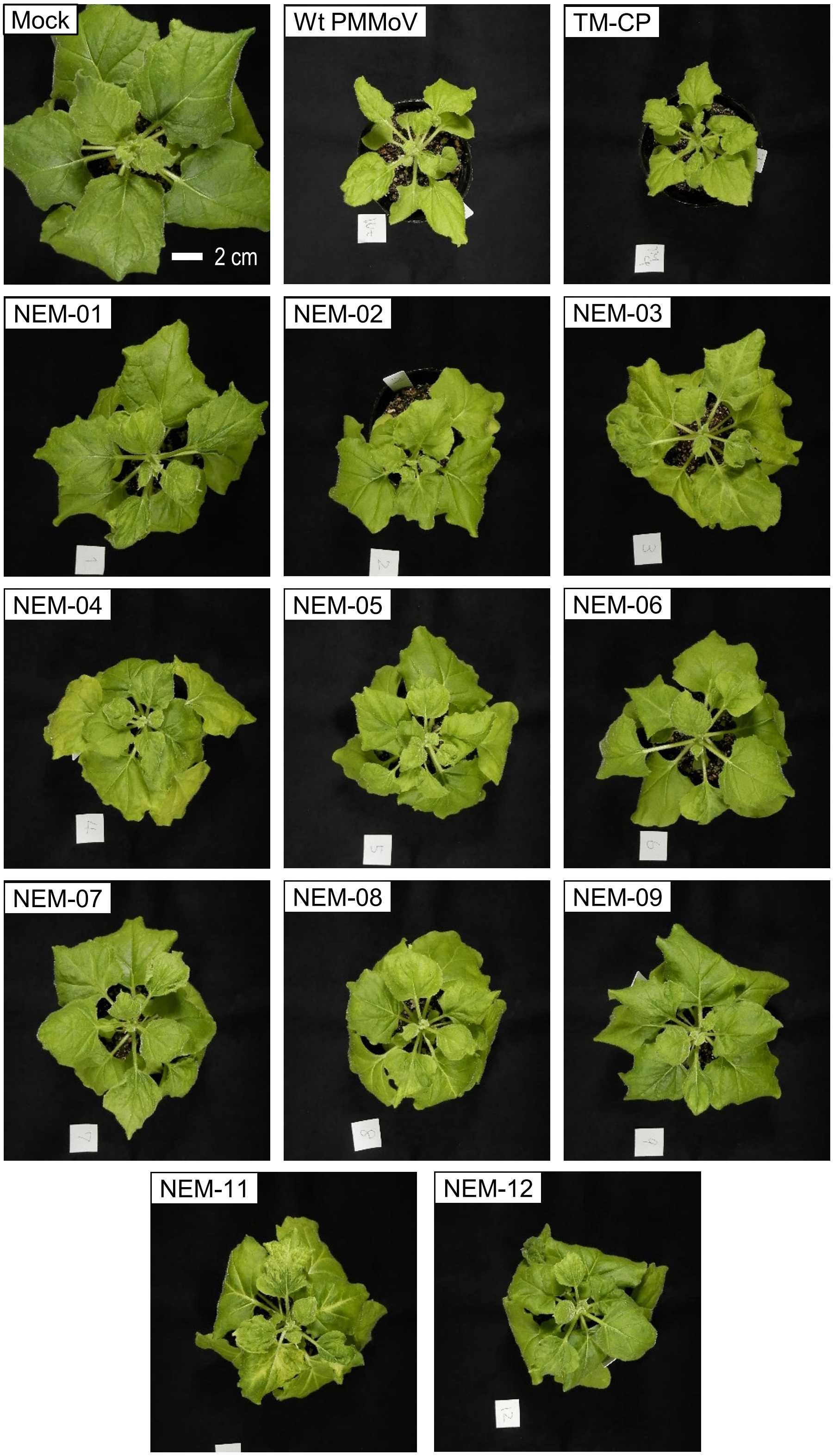

Continues to the next page

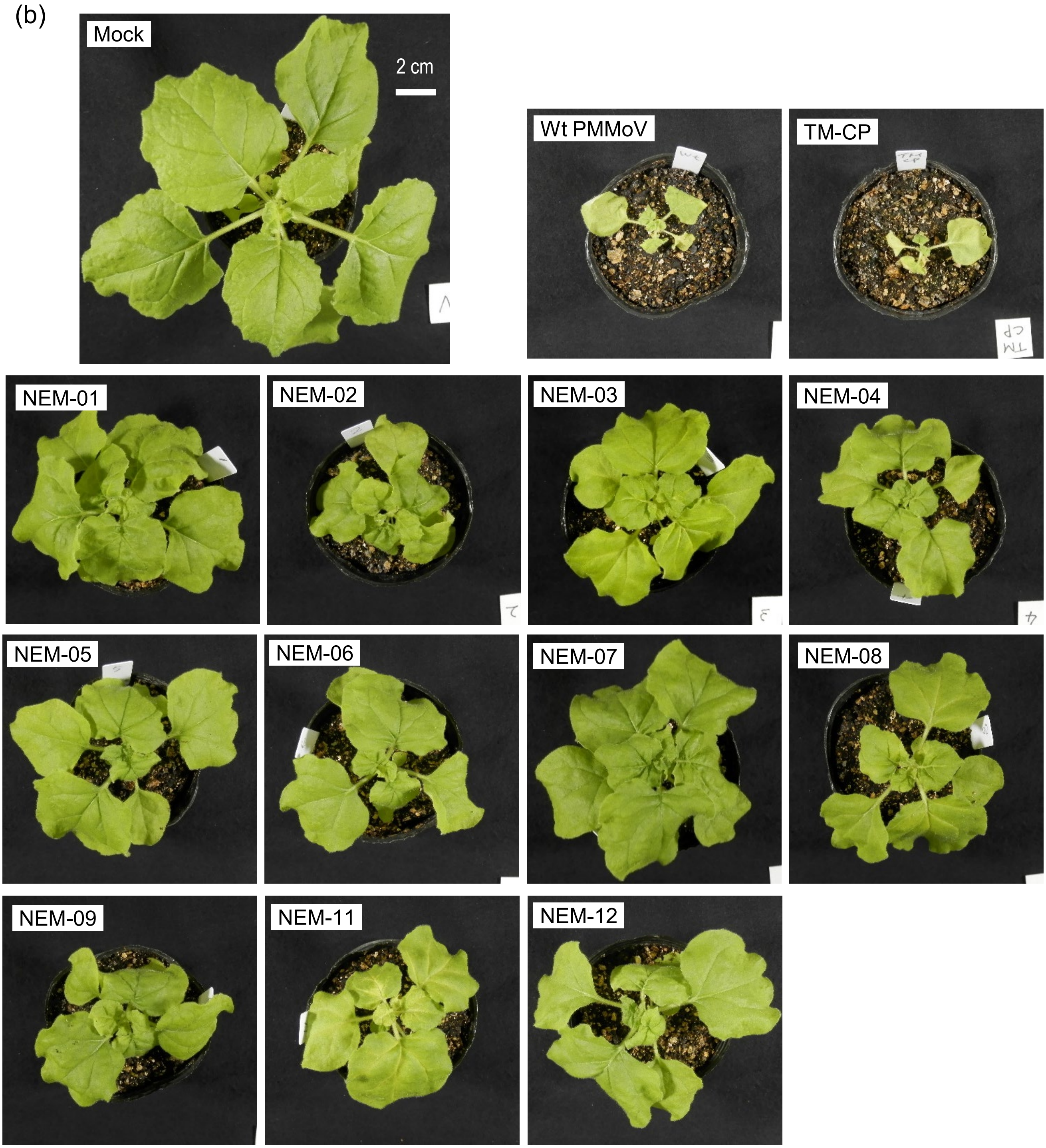

Supplementary Figure S7. Systemic infection of *N. benthamiana* with *N*´-escaping mutants (NEMs). *N. benthamiana* leaves were surface-inoculated with wild-type (WT), PMMoV with TMV CP (TM-CP), and NEMs PMMoV clones. Plants were inoculated at 3 weeks post-seed sowing (a) or 2 weeks post-seed sowing (b) and photographed 3 weeks post-inoculation.

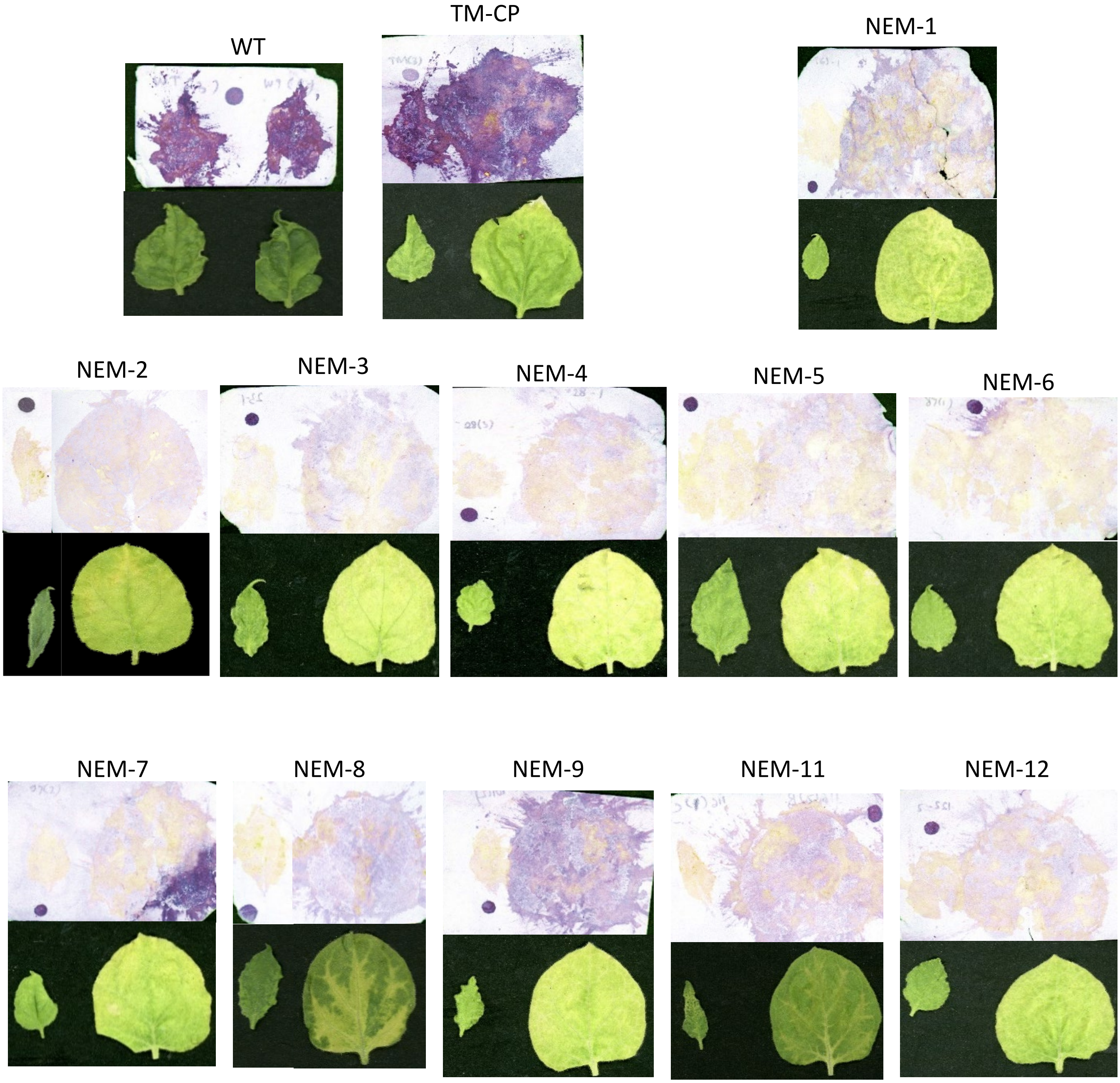

Supplementary Figure S8. Age-dependent accumulation of *N*´-escaping mutants (NEMs) in systemically infected *N. benthamiana*. *N. benthamiana* leaves were surface-inoculated with wild-type (WT) and NEMs PMMoV clones. Plants were inoculated at 3 weeks post-seed sowing, and different leaves were analyzed at 5 weeks post-inoculation.

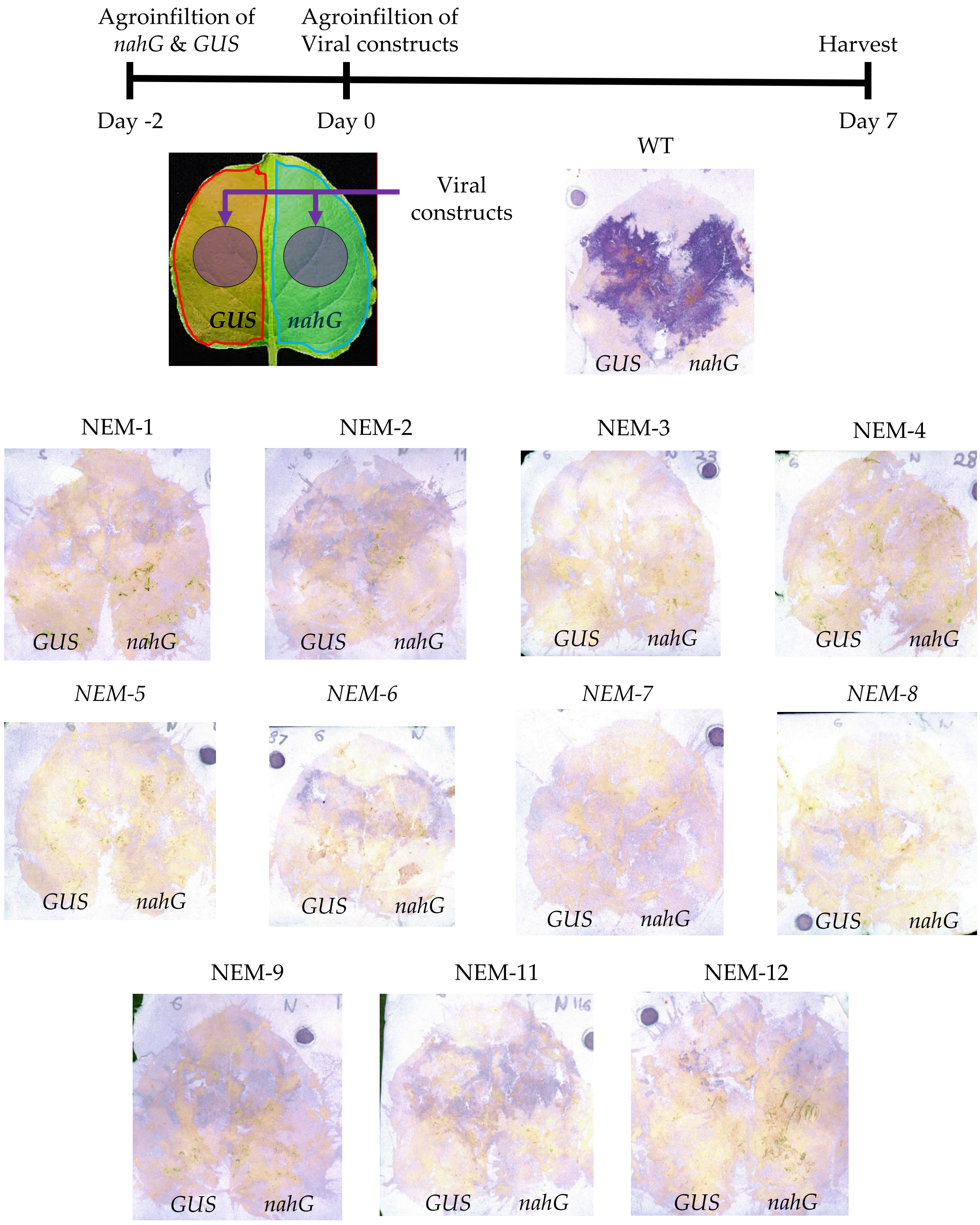

Supplementary Figure S9. Effect of salicylate hydrolase gene expression on the accumulation of *N*´-escaping mutants (NEMs) in agroinfiltrated *N. benthamiana* leaves. *N. benthamiana* leaves were agroinfiltrated with constructs expressing salicylate hydrolase gene (*nahG*) and β-glucuronidase gene (*GUS*) as a control at a bacterial density of 0.2. They were agroinfiltrated 2 days later with wild-type (WT) and NEMs PMMoV clones at a bacterial density of 0.01. Virus accumulation was evaluated by hammer blot immunoassay at 7 days post-infection.

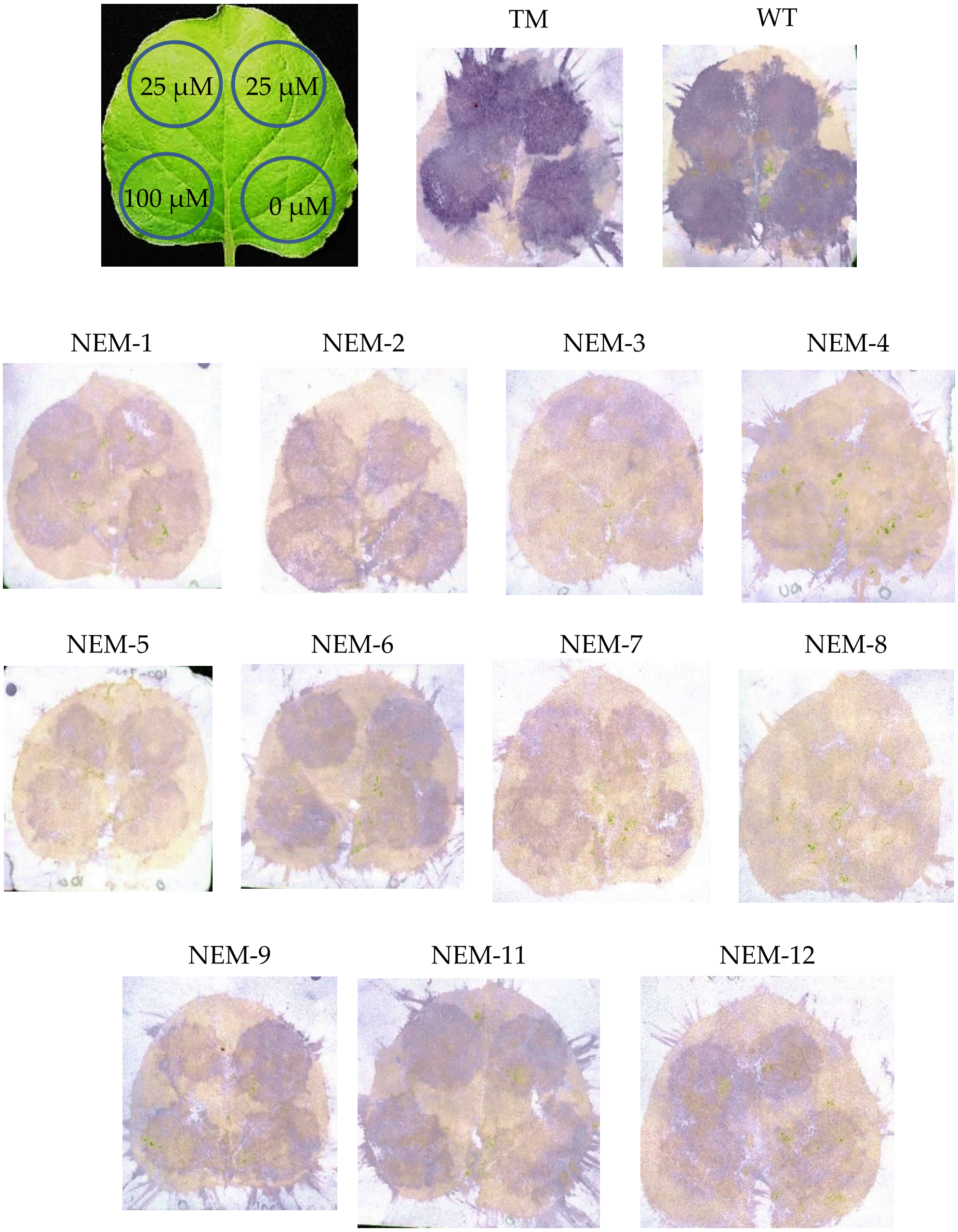

Supplementary Figure S10. Effect of salicylic acid signaling inhibitor on the accumulation of *N* ´-escaping mutants (NEMs) in agroinfiltrated *N. benthamiana* leaves. *N. benthamiana* leaves were infiltrated with wild-type (WT) and NEMs PMMoV clones at a bacterial density of 0.01 without (0 µM) or with Tenoxicum (TNX) at the indicated concentration. Virus accumulation was evaluated by hammer blot immunoassay at 7 days post-infection.

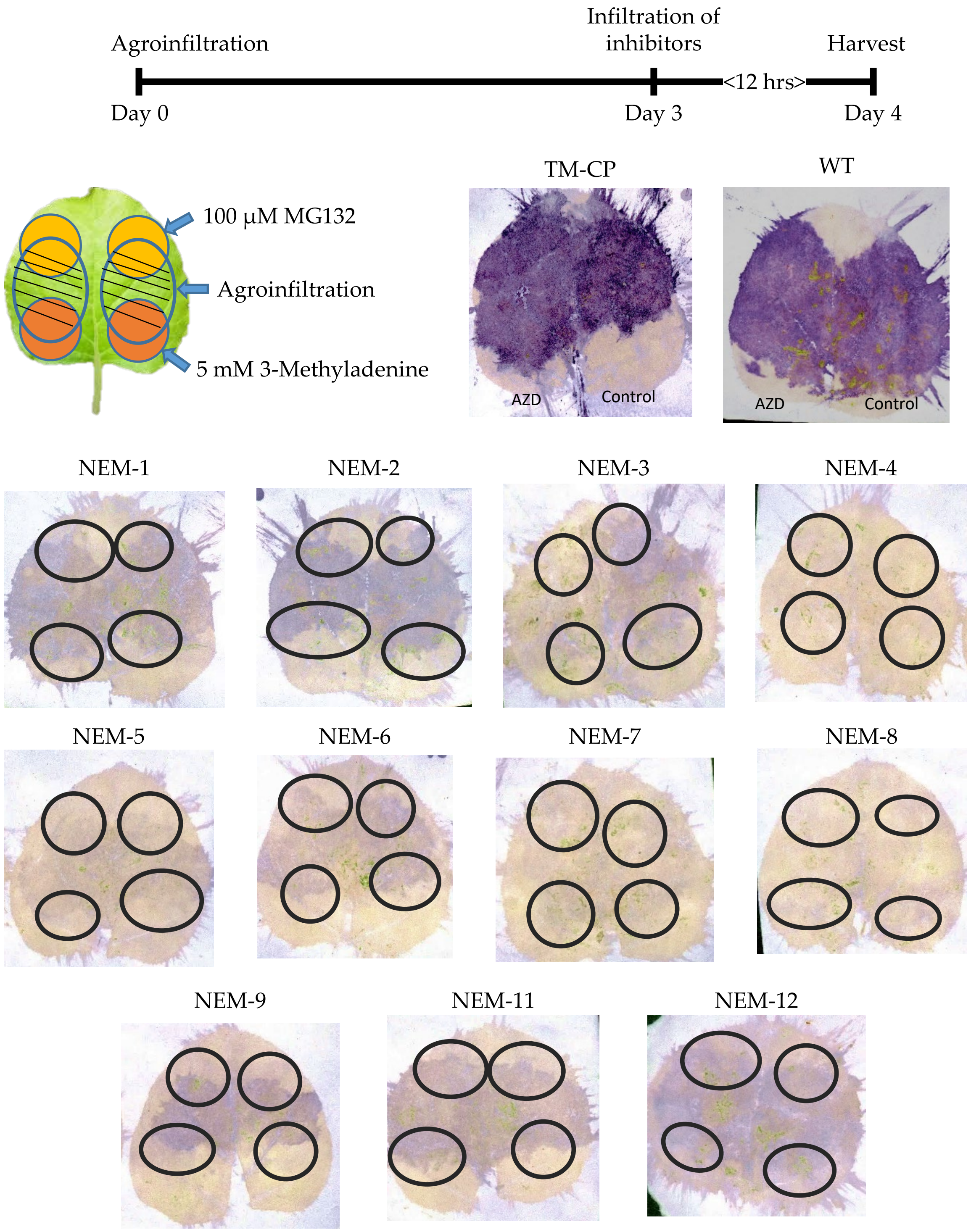

Supplementary Figure S11. Effect of proteasome or autophagy inhibitors on the accumulation of *N*´-escaping mutants (NEMs) in agroinfiltrated *N. benthamiana* leaves. *N. benthamiana* leaves were infiltrated with wild-type (WT) and NEMs PMMoV clones at a bacterial density of 0.01. After 3 days, parts of the agroinfiltrated areas were infiltrated with 100 µM MG132 or 5 mM 3-Methyladenine. Virus accumulation was evaluated by hammer blot immunoassay after 12 hrs of the inhibitor treatments.
