# Supplementary material for: A Tradeoff Between the Escape from N′-Mediated Resistance and Virulence in Pepper Mild Mottle Virus Through Reduced Virus Accumulation
Source: Plants (Basel). 2025 Aug 9;14(16):2471. doi: 10.3390/plants14162471 (PMC12389172; doi:10.3390/plants14162471)
Supplement: Supplementary file 1 [file plants-14-02471-s001.zip › Supplementary_table S1-R1&S2.pdf]

Supplementary Table S1. Summary of analyses of *N´*-escaping mutants and their derivatives

Name[a]	Amino acid substitutions[b]	HR[c]	Inoc[d]	Accum[e]	Pepper[f]
NEM-01	S5P L23I T54A P63S I94V N140S	0	18	+	NT
1a*	S5P L23I T54A P63S	0	12	+	-
1b	I94V N140S	9	12	++	NT
1c	S5P L23I	12	12	++	NT
1d	T54A P63S	10	12	+	NT
1e	S5P L23I T54A	7	12	++	NT
1f	P63S	10	12	+	NT
1g	L23I P63S	12	12	+++	NT
1h	S5P P63S	5	12	+	NT
1i	L23I T54A P63S	5	6	NT	NT
1j	S5P T54A P63S	3	6	NT	NT
1k	S5P L23I P63S	2	6	NT	NT
1l	S5P T54A	4	6	NT	NT
1m	L23I T54A	6	6	NT	NT
1n	T54A	2	6	NT	NT
1o	L23I	6	6	NT	NT
1p	S5P	6	6	NT	NT
NEM-02	L13I R41S K68N F87L I125M	0	18	++	-
2a	L13I R41S K68N	0	12	+	-
2b	F87L I125M	7	12	+	NT
2c	L13I	7	12	+++	NT
2d	R41S K68N	7	12	+	NT
2e	L13I R41S	8	12	+	NT
2f	K68N	1	12	+++	NT
2g*	L13I K68N	0	12	-	NT
NEM-03	Y12C F35S Q45R N73S S148R	0	18	+	NT
3a	Y12C F35S Q45R N73S	0	12	+++	-
3b	S148R	10	12	+	NT
3c	Y12C	4	6	+	NT
3d	F35S Q45R N73S	7	12	+	NT
3e	Y12C F35S Q45R	4	12	+	NT
3f	N73S	8	12	++	NT
3g	Y12C N73S	7	12	++	NT
3h	Y12C Q45R N73S	4	6	NT	NT
3i	Y12C F35S N73S	5	15	NT	NT
3j*	Y12C Q45R	0	9	-	NT
3k	Q45R N73S	5	6	NT	NT
3l	F35S Q45R	10	18	NT	NT
3m	F35S N73S	3	6	NT	NT
3n	Y12C F35S	2	6	+	NT
3o*	F35S	0	18	-	NT
3p	Q45R	4	6	+	NT

NEM-04	N25D C27R Q36H V51A F70S M129K	0	18	+	NT
4a	N25D C27R Q36H V51A F70S	0	12	+	-
4b	M129K	9	12	+	NT
4c	N25D C27R	0	12	+	NT
4d	Q36H V51A F70S	3	12	+	NT
4e	N25D C27R Q36H V51A	0	12	+	NT
4f	F70S	11	12	+	NT
4g	N25D	9	18	++	NT
4h*	C27R	0	12	+	-
NEM-05	Y12S F62N	0	18	+	NT
5a	Y12S	5	12	+	NT
5b*	F62N	0	12	+	-
NEM-06	T57S T103S R122G Y139D	0	18	+	NT
6a	T57S	8	15	+++	NT
6b	T103S R122G Y139D	0	12	+	-
6c	T103S	8	12	+++	NT
6d	R122G	6	12	+	NT
6e*	Y139D	0	12	-	NT
6f	T103S R122G	10	12	+	NT
6g	T103S Y139D	3	12	+	NT
6h	R122G Y139D	0	12	-	NT
NEM-07	C27R N130I	0	18	+	NT
7a*	C27R	0	12	+	-
7b	N130I	8	12	++	NT
NEM-08	N101D L128H F144Y W152R	0	18	+	NT
8a	N101D	5	6	+++	NT
8b	L128H	2	6	+	NT
8c	F144Y	5	12	++	NT
8d	W152R	6	12	+	NT
8e	N101D L128H	1	6	+	NT
8f	N101D F144Y	6	6	+	NT
8g	N101D W152R	5	6	-	NT
8h*	L128H F144Y	0	12	-	NT
8i*	L128H W152R	0	12	-	NT
8j*	F144Y W152R	0	12	+	-
8k	N101D L128H F144Y	0	12	+++	-
8l	N101D L128H W152R	0	12	+	-
8m	N101D F144Y W152R	0	12	++	-
8n	L128H F144Y W152R	0	12	-	NT
NEM-09	N25H Q38R Q46P Q141H	0	18	+	-
9a	N25H Q38R Q46P	6	11	+	NT
9b	Q141H	11	12	++	NT
9c	N25H	10	16	+++	NT
9d	Q38R Q46P	2	12	++	NT
9e	N25H Q141H	5	6	+	NT

9f	Q38R Q141H	5	6	++	NT
9g*	Q46P Q141H	0	12	-	NT
9h	Q46P	3	6	NT	NT
NEM-11	Y2N N8K S49P S78P V114A	0	18	++	NT
11a	Y2N N8K S49P S78P	0	12	+	NT
11b	V114A	12	12	+++	NT
11c	Y2N N8K	3	12	+	NT
11d	S49P S78P	8	12	+	NT
11e	Y2N N8K S49P	0	12	+	NT
11f	S78P	9	12	++	NT
11g	N8K S78P	3	12	++	NT
11h	Y2N S78P	1	6	+	NT
11i*	N8K S49P	0	12	-	NT
11j*	Y2N S49P	0	12	+	-
11k	Y2N	7	18	NT	NT
11l	N8K	3	6	NT	NT
11m	S49P	5	6	NT	NT
NEM-12	F35L R41G F62L F70S	0	18	++	NT
12a	F35L R41G	6	12	++	NT
12b*	F62L F70S	0	12	+	-
12c	F62L	13	18	NT	NT
12d	F70S	11	12	NT	NT

[a] Name of NEMs or derivatives. Asterisks denote the derivative with minimal mutations.

[b] Amino acid substitutions in each NEM or derivative

[c] Number of inoculation spots exhibiting hypersensitive reaction

[d] Number of spots with toothpick inoculation

[e] Accumulation of CP in agroinfiltrated *N. benthamiana* ; -, undetected; +++, comparable to the wild type; ++, slightly lesser than the wild type; +, significantly lesser than the wild type; NT, not tested.

[f] Systemic infection of pepper; -, undetected; NT, not tested.

Supplementary Table S2. Primers used in this study

Primer name	Sequence	PCR condition and other comments	
Primers for the detection and sequencing of CP genes			
PMF1	GTTAGAATTGGGCAGAACTCGG	94°C, 1 min; 25 cycles of 94°C for 30 s, 60°C for 30 s, and 72°C for 1 min; and 72°C for 3 min, with Taq DNA polymerase	
PMR1	GTTATCGTACTCGCCACGGA		
Quantitative PCR primers			
PMMoVqP02F	CAACTGCAGAGGTGGAGTAAG	These were used according to the manufacturer's specifications for the quantitative PCR kit.	
PMMoVqP02R	TTAGCCGCACTGGTTCTATAAG		
Nico18SqP-F	GGTGGAGCGATTTGTCTGGT		
Nicol18qP-R	CAGGCTGAGGTCTCGTTCGT		
Primer Sequences for nahG Cloning and Vector Confirmation			
pRI_Xb_A65nahG-F	GATATGTTATGTATGTGCAGggtaccatgaaaaacaataaacttgg	94°C, 2 min; 25 cycles of 95°C for 10 s, 50°C for 0.05 s, and 68°C for 1 min; and 68°C for 7 min, with PrimeSTAR GXL DNA polymerase	
nahG-pRI_SacIR	CTTCATCTTCATAAgagctctcacccttgacgtagcgcacc		
Upro-835F	CTGGACTTTTTGGAGTTGTTGAC	Sequencing primer	
HSPter-SR	GCCACAAATTCATAACACAACA		
nahG_qRT_679F	CTCGACGGCCATATCCTCAC		
nahG_qRT_749R	TCGGAGATGAAAGCCACCAC		
Primers for minimal mutations search			
PMCPF91	ACTTCGGCGTTAGGTAATCA	Paired with PMCPF175, PMCPR245, or PMR1	1st PCR: 94°C, 2 min; 25 cycles of 95°C for 10 s, 50°C for 5 s, and 68°C for 30 sec; and 68°C for 7 min, with PrimeSTAR GXL DNA polymerase<br>2nd PCR (Recombinant PCR): 1st PCR: 94°C, 2 min; 25 cycles of 95°C for 10 s, 50°C for 5 s, and 68°C for 1 min; and 68°C for 7 min, with PrimeSTAR GXL DNA polymerase
PMCPR91	TGATTACCTAACGCCGAAGT	Paired with PMF1	
PMCPF175	CCGACCGCTACAGTTAGATT	Paired with PMCPR245 or PMR1	
PMCPR175	AATCTAACTGTAGCGGTCGG	Paired with PMF1, or PMCPF91	
PMCPF245	TAGTGTCGGCACTTCTCGGAGCCTT	Paired with PMR1	
PMCPR245	AAGGCTCCGAGAAGTGCCGACACTA	Paired with PMF1, PMCPF91, or PMCPF175	

S6P-F	TACACAGTTCCCAGTGCCAATCAA	Primers for site-derected mutagenesis, which were paired with PMR1/PMR1, or other appropriate primers
S6P-R	TTGATTGGCACTGGGAACTGTGTA	
L24I-F	TCCATTAGAGATACAAAATCTATGTA	
L24I-R	TACATAGATTTTGTATCTCTAATGGA	
K69N-F	TACTGGTTTCAATGTTTTTCGATAT	
K69N-R	ATATCGAAAAACATTGAAACCAGTA	
Y3N-F	TAACACAGTTTCCAGTGCCAATCAA	
Y3N-R	TTGATTGGCACTGGAAACTGTGTTA	
N9K-F	TTACACAGTTTCCAGTGCCAAACAATT	
N9K-R	AATTGTTTGGCACTGGAAACTGTGTAA	
S104T-F	AAAATCCTACAACTGCCGAGACGCT	
S104T-R	AGCGTCTCGGCAGTTGTAGGATTTT	
R123G-F	ATTGGGGCCAGTATAAGTAA	
R123G-R	TTACTTATACTGGCCCCAAT	
G123R-F	ATTAGGGCCAGTATAAGTAA	
G123R-R	TTACTTATACTGGCCCTAAT	
D140Y-F	AATGTACAATCAAGCTCTGTTCGA	
D140Y-R	TCGAACAGAGCTTGATTGTACATT	
L129H-F	ATAAGTAACCACATGAATGA	
L129H-R	TCATTCATGTGGTTACTTAT	
F145Y-F	AATCAAGCTCTGTACGAGA	
F145Y-R	TCTCGTACAGAGCTTGATT	
H129L-F	ATAAGTAACCTCATGAATGA	
H129L-R	TCATTCATGAGGTTACTTAT	
N102D-F	AAATCCGCAAGATCCTACAA	
N102D-R	TTGTAGGATCTTGCGGATTT	
D102N-F	AAATCCGCAAAATCCTACAA	
D102N-F	TTGTAGGATTTTGCGGATTT	
N25H-F	TTACAACATCTATGTACTT	

N25H-R	AAGTACATAGATGTTGTAA		
Q38R-F	AATCAGTTTCAAACACGGCA		
Q38R-R	TGCCGTGTTTGAAACTGATT		
Q46P-F	TTCAACCGCAGTTCTCTGAT		
Q46P-R	ATCAGAGAACTGCGGTTGAA		
F62L-F	TCAAAGTTTCTCGATATAAT		
F62L-R	ATTATATCGAGAAACTTTGA		
T54S-F	TGATGTGTGGAAGGCCATA		
T54S-R	TATGGCCTTCCACACATCA		
F35S-F	AGGTAATCAGTCTCAAACA		
F35S-R	TGTTTGAGACTGATTACCT		
Q45R-F	AACTACGGTTCGACAGCAG		
Q45R-R	CTGCTGTCGAACCGTAGTT		
